# GluD1 is localized at cholinergic synapses and is an acetylcholine receptor

**DOI:** 10.1038/s41380-026-03675-4

**Published:** 2026-06-10

**Authors:** Poojashree B. Chettiar, Kishore Kumar S. Narasimhan, Siddhesh S. Sabnis, Spencer S. Ericksen, Diane Choi, Yoland Smith, Shashank M. Dravid

**Affiliations:** 1 Department of Psychiatry and Behavioral Sciences, Naresh K. Vashisht College of Medicine, Texas A&M University, 206, Olsen Blvd, Reynolds Medical Sciences Bldg., College Station, TX 77845 USA; 2 Drug Development Core, Small Molecule Screening Facility, University of Wisconsin, Carbone Cancer Center, University of Wisconsin-Madison, Madison, WI USA; 3 Emory National Primate Research Center, Emory University, Atlanta, GA USA; 4 Department of Neurology, Emory University, Atlanta, GA USA

**Keywords:** Neuroscience, Physiology, Autism spectrum disorders

## Abstract

The GluD1 receptor has many unusual features including expression at both excitatory and inhibitory synapses and a ligand binding domain capable of binding both D-serine and GABA. We have previously demonstrated that striatal GluD1 is critical for the regulation of behavioral flexibility, a phenotype dependent on the cholinergic system. Here, we found that GluD1 is enriched postsynaptically at cholinergic synapses in the mouse and monkey dorsal striatum. Further, loss of GluD1 reduces the abundance of cholinergic terminals, excitatory responses at cholinergic synapses as well as muscarinic receptor-induced plasticity. In addition, optogenetic stimulation of cholinergic interneurons or puff-application of ACh, in the presence of cholinergic and AMPA/GABA_A_ receptor blockers, produced current responses in medium spiny neurons (MSNs) that were sensitive to the GluD1-channel blocker NASPM. These responses were absent in GluD1 KO and overexpression of GluD1 on KO background rescued Ach puff-induced currents suggesting potential conductance via GluD1. Finally, using GluD1-Cbln1 interaction assay as an indirect method to evaluate ligand binding interaction, we found that ACh can bind GluD1 and induce conformational changes. A similar ACh-induced conformational change was observed for GluD2 in the cell binding assay. Importantly, molecular dynamics simulations and mutagenesis analysis demonstrated that ACh binding orientation in the GluD1 ligand binding domain is different from D-serine and GABA. Overall, our results identified an unprecedented feature of GluD1 in the regulation of cholinergic synapses.

## Introduction

The ionotropic glutamate receptors (iGluRs) family consists of AMPA, kainate, NMDA and delta receptors. Glutamate delta receptors (GluDs) are unique within the ionotropic glutamate receptor (iGluR) family, as they do not exhibit typical ligand-gated ion channel activity in heterologous expression systems. Nevertheless, endogenous neurotransmitters such as D-serine, glycine, and GABA can bind to their ligand-binding domain (LBD) and induce conformational changes in the receptor [1–5]. Consistent with this, a recent study demonstrated that D-serine and GABA induce ion channel activity in GluD2 in a lipid bilayer system, but not when expressed in cells [6]. However, ligand binding to GluDs in native system facilitates synaptic plasticity. For instance, D-serine binding to GluD2 contributes to long-term depression (LTD) at parallel fiber-Purkinje cell synapses during early development [7], whereas GABA binding to GluD1 facilitates plasticity at inhibitory synapses [3]. Furthermore, various studies have established the role of GluDs in synaptogenesis. GluDs-cerebellins (Cblns)-neurexins form a trans-synaptic complex and facilitate synapse formation and maintenance [8–10]. Consistent with this, GluD1 is obligatory for the formation/maintenance of excitatory and inhibitory synapses in specific circuits [11–14]. Loss of GluD1 leads to a reduction in excitatory neurotransmission in the dorsal striatum medium spiny neurons (MSNs) and laterocapsular central amygdala neurons and inhibitory neurotransmission in the somatosensory cortex and nucleus accumbens [10, 15–18].

We have previously found that GluD1 in the dorsal striatum is critical for the regulation of cognitive behavioral flexibility [15], a phenotype highly dependent on the cholinergic system [19–21]. Cholinergic interneurons in the dorsal striatum serve as a primary source of acetylcholine (ACh), which modulates glutamatergic and GABAergic transmission and regulates the balance between the direct and indirect stratiofugal pathways. Cholinergic modulation is crucial for action–outcome associations and the suppression of previously learned responses, which are essential for adaptive behavioral flexibility [19–21]. Therefore, to understand the relationship between GluD1 and the cholinergic system, we examined the localization of GluD1 at cholinergic synapses and its potential role in regulating cholinergic synapses function and ionic conductances at these synapses. We found that GluD1 is localized at cholinergic synapses and that loss of GluD1 results in a reduced prevalence of cholinergic terminals and changes in muscarinic receptor-mediated plasticity. Furthermore, either optogenetic stimulation of cholinergic interneurons or puff application of ACh in the presence of GABA_A_, AMPA and cholinoreceptor inhibitors resulted in current responses in MSNs that were sensitive to the GluD1 blocker NASPM and largely absent in GluD1 KO. We also found that ACh binds to both GluD1 and GluD2 and induces conformational changes in an assay based on GluD1/GluD2-Cbln1 interaction [22]. We found that ACh binds to both GluD1 and GluD2 and induces conformational changes. Together, these results identify a novel and unprecedent role of GluD1 in regulating cholinergic transmission.

## Materials and methods

All animal and other experiments were performed in accordance with NIH guidelines and regulations at Texas A&M University and Emory University.

### Animals

All animal procedures were approved by the Texas A&M University and Emory University Institutional Animal Care and Use Committees (IACUC). Two to four months old, male and female wildtype (WT), GluD1 knockout (KO) and striatum specific knockout (St-GluD1 KO) [15] mice were used. The monkey tissue used to collect immuno-EM data came from three rhesus monkeys (2F, 1M; 5–12 years old) from the Emory National Primate Research Center colony. The antibodies, chemicals, drugs used in this study are listed in the Resources Table S2.

### Stereotaxic surgery and AAV injections

Stereotaxic surgery was conducted as previously described [23]. A small hole was made at the coordinates targeting the dorsal striatum. A glass injection needle was then lowered to the target site, and viral particles were infused at a rate of 1 nl/s.

### Immunohistochemistry

Imaging was done using a Nikon Ti2 confocal microscope with a 60× oil immersion objective. Puncta number and co-localization were quantified using NIS-Elements software [15].

### Immuno-electron microscopy

Sections were processed for double immunolabeling with guinea-pig anti-vAChT (immunoperoxidase) and rabbit anti-GluD1 (pre-embedding immunogold) using techniques previously described [24].

### Synaptoneurosome preparation and immunoblotting

Brain tissue was homogenized in synaptoneurosome isolation buffer and sequentially filtered followed by centrifugaton. SDS-PAGE resolved equal protein amounts, transferred to PVDF membranes, and probed with primary antibodies.

### Brain slice electrophysiology

Whole-cell recordings from MSNs in the dorsal striatum were performed as described [15] using an Axopatch 200B amplifier with Cs-based internal solution containing QX-314. Cells were voltage-clamped at –70 mV or other voltages at indicated and responses were evoked by optogenetically or by puff ACh application (1 mM, 50 ms, 8 psi) or baseline excitatory neurotransmission was measured.

### Cell culture, transfection and cell binding assay

HEK293T or COS-7 cells were transfected with pCAGGS rat GluD1 WT, E446Q, or R526K or GluD2 WT using PEI. For quantitative binding assays, HEK293T cells were incubated with His-tagged recombinant Cbln1 (5 µg/mL) for 4 h following 30 min ligand or vehicle treatment [22]. Cell lysates were collected, and immunoblotted with anti-His, anti-GluD1, and anti-β-actin antibodies. For ICC-based binding assays, transfected COS-7 cells were treated with HA-tagged Cbln1 (1 µg/mL) for 4 h, fixed with 4% PFA, blocked with 3% BSA, and incubated with anti-HA primary and AlexaFluor 488–conjugated secondary antibodies, mounted and imaged using a Nikon Ti2 confocal microscope [22].

### MD simulations

All systems were parameterized with CHARMM36 and CGenFF force fields, solvated in TIP3P water with 0.15 M NaCl, and simulated in GROMACS 2024.4 under NPT conditions (310 K, 1 bar) with 2 fs time steps. After staged equilibration with positional restraints on protein Cα atoms, 200 ns production runs were performed.

### Statistical analysis

Data was analyzed in GraphPad Prism 8.0. Two-group comparisons used unpaired two-tailed *t*-tests; multi-group comparisons used one-way ANOVA with Sidak’s post hoc test (*p* < 0.05).

## Results

### GluD1 is localized at cholinergic terminals

**Fig. 1:**
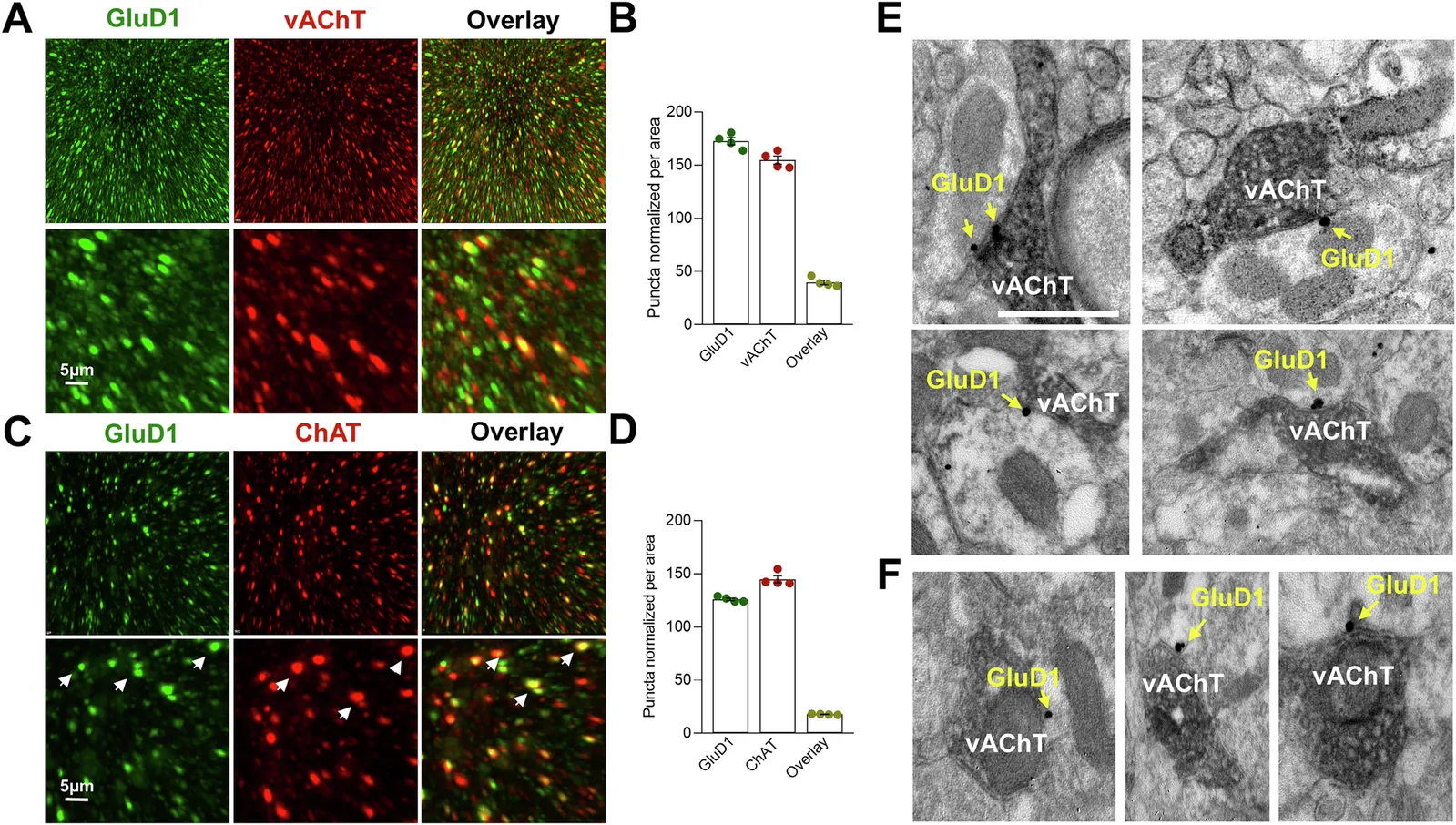
GluD1 localizes to cholinergic terminals. **A** Immunofluorescent labeling of GluD1 (green) and vesicular acetylcholine transporter (vAChT, red) in the mouse dorsal striatum reveals frequent puncta co-localization (arrows) suggesting GluD1 expression at cholinergic terminals. **B** Corresponding quantification of puncta (normalized to representative field) for GluD1, vAChT and its co-localization. Data points represent the average of images analyzed from individual animals (*N* = 4 animals). Scale bar: 5 μm. **C** Immunolabeling of GluD1 (green) and choline acetyltransferase (ChAT, red) also shows co-localized puncta further supporting GluD1’s presence at cholinergic terminals in the striatum. **D** Quantification of the puncta shows co-localization of GluD1 and ChAT. Each data point represents the average of images analyzed from individual animals (*N* = 4 animals). Scale bar: 5 μm. **E** Electron micrographs showing GluD1 immunogold labeling (yellow arrows) at symmetric synapses formed by VAChT+ terminals (immunoperoxidase labeling) in the mouse dorsal striatum (*N* = 2 mice; 2 tissue blocks/mouse; 50 micrographs/block). Scale bar: 0.5 μm (valid for F). **F** Electron micrographs showing GluD1 immunogold labeling (yellow arrows) at symmetric synapses formed by vAChT+ terminals (immunoperoxidase labeling) in the monkey caudate nucleus.

We first examined the localization of GluD1 in the mouse dorsal striatum using immunohistochemical techniques. At the light microscopy level, GluD1 immunoreactivity is robustly detected in the striatum of WT mice, absent in the GluD1 KO mice and selectively absent in the St-GluD1 KO validating the specificity of the GluD1 antibody in the striatum (Fig. S1A). Double-immunolabeling studies for GluD1 and cholinergic markers, vesicular acetylcholine transporter (vAChT) or choline acetyl transferase (ChAT), showed strong colocalization of GluD1 puncta with vAChT and ChAT in the striatum (Fig. 1A–D). We also examined GluD1 expression in the interpeduncular nucleus (IPN), which receives cholinergic projection from Cbln4-positve medial habenula neurons [25] and found GluD1 colocalized with vAChT (Fig. S1B). These results demonstrate that GluD1 is enriched at cholinergic terminals where it may influence ACh-dependent signaling in mice. To further support these observations, we probed the ultrastructural localization of GluD1 and its relationship with striatal cholinergic synapses using double-immuno EM. In both the mouse and monkey dorsal striatum, GluD1 immunogold particles were found at immunoperoxidase-labeled vAChT-positive synapses (Fig. 1E, F). In addition, GluD1 was also found to be expressed at vAChT-positive synapses in the monkey IPN (Fig. S1C-D). Together, these results highlight a conserved localization of GluD1 at cholinergic synapses in both rodents and non-human primates.

### GluD1 is critical for the maintenance of striatal cholinergic synapses

**Fig. 2:**
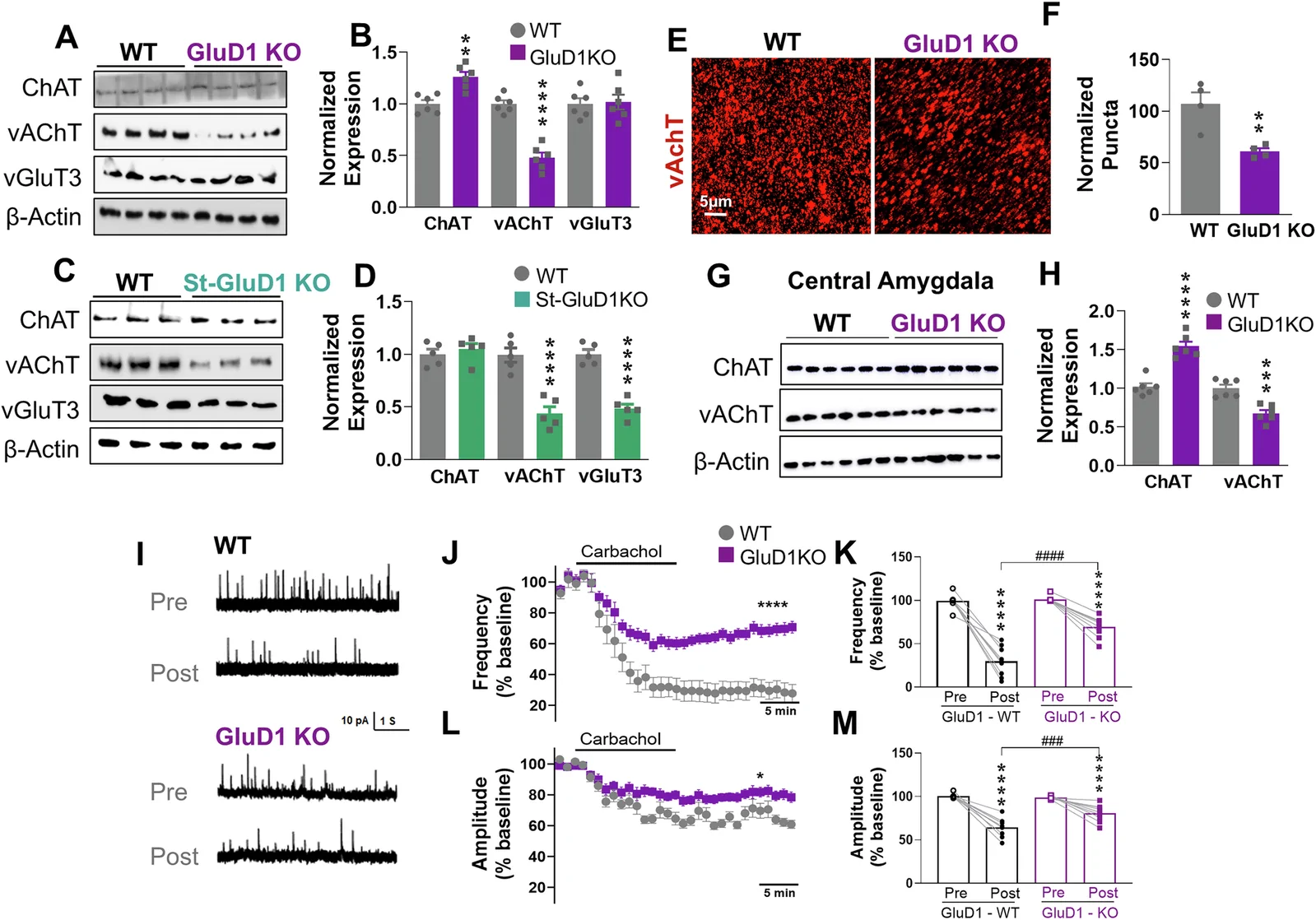
Regulation of abundance of striatal cholinergic terminals by GluD1. **A** Immunoblot analysis of synaptoneurosome preparations from the dorsal striatum of wild-type and global GluD1 knockout (KO) mice, probing for choline acetyltransferase (ChAT), vesicular acetylcholine transporter (vAChT), and vesicular glutamate transporter 3 (vGluT3). A significant downregulation of vAChT and an upregulation of ChAT were observed in GluD1 KO mice, while vGluT3 levels remained unchanged. **B** Densitometric quantification of immunoblots shows a significant increase in ChAT (**P = 0.0027) and a decrease in vAChT (****P < 0.0001). **C** Immunoblot analysis of synaptoneurosome preparations from the dorsal striatum of wild-type and striatal GluD1 conditional knockout (cKO) mice. In contrast to the global KO, vAChT and vGluT3 levels were significantly reduced in the GluD1 cKO mice, while ChAT levels remained unchanged. **D** Corresponding densitometric analysis confirms significant downregulation of vAChT and vGluT3 (****P < 0.0001). **E** Representative confocal images of immunohistochemical staining for GluD1 and vAChT in the dorsal striatum of wild-type and GluD1 KO mice. A marked reduction in vAChT puncta was observed in GluD1 KO. **F** Quantification of vAChT puncta from 30–35 images per group (*n* = 4 mice; 4–6 sections per mouse) revealed a significant decrease in vAChT puncta in GluD1 KO mice (**P = 0.007). Unpaired t-test. **G** Immunoblot analysis of synaptoneurosome preparations from the central amygdala of wild-type and global GluD1 knockout (KO) mice, probing for cholinergic markers ChAT and vAChT. A significant upregulation of ChAT and a downregulation of vAChT was observed in GluD1 KO mice. **H** Densitometric quantification shows a significant increase in ChAT (***P < 0.0001) and a decrease in vAChT (***P = 0.0003). Data are presented as mean ± SEM; analyzed using umpaired t-tests with Welch’s correction (*N* = 6 mice per group). **I** Representative traces of NMDA receptor–mediated miniature excitatory postsynaptic currents (NMDA-mEPSCs) recorded from MSNs in WT and GluD1 knockout (KO) conditions before (Pre) and after (Post) bath application of muscarinic agonist carbachol (5 µM). In WT neurons, carbachol produced a reduction in NMDA-mEPSC frequency and amplitude, whereas this effect is diminished in GluD1 KO neurons. **J** Time course of normalized NMDA-mEPSC frequency (% baseline) following bath application of carbachol (shaded region). WT neurons exhibit a rapid and sustained decrease in event frequency following muscarinic activation. In contrast, this depression is significantly attenuated in GluD1 KO neurons (*N* = 9; ***P < 0.0001). **K** Summary quantification of NMDA-mEPSC frequency before (Pre) and after (Post) carbachol application. WT neurons display robust depression of NMDA-mEPSC frequency, whereas GluD1 KO neurons show significantly reduced suppression. Lines connect paired recordings from individual neurons (***P < 0.0001 for Pre vs Post; ^####^*P* < 0.0001 for WT post vs GluD1 KO post). **L** Time course of normalized NMDA-mEPSC amplitude (% baseline) following carbachol application (shaded region). Muscarinic activation reduces NMDA-mEPSC amplitude in WT neurons, consistent with postsynaptic NMDAR depression. This effect is partially preserved but significantly attenuated in GluD1 KO neurons (*P = 0.0400). **M** Summary quantification of NMDA-mEPSC amplitude before and after carbachol application. WT neurons exhibit a significant reduction in amplitude, whereas the magnitude of depression is reduced in GluD1 KO neurons (***P < 0.0001 for Pre vs Post; ^###^*P* = 0.0002 for WT post vs GLuD1 KO post).

Given the critical role of GluD1 in synapse maintenance [10, 15, 16], we explored whether GluD1 is obligatory for the maintenance of cholinergic synapses in the dorsal striatum. Using immunoblotting, we examined potential changes in markers of cholinergic terminals in the synaptoneurosome preparation from dorsal striatum of WT and GluD1 KO mice. A significant reduction in the expression of vAChT and no change in vGluT3 was observed in striatal synaptoneurosomes from GluD1 KO mice (Fig. 2A, B). Interestingly an increase in ChAT expression was observed. We also assessed the expression of vAChT and vGluT3 in conditional KO model in which GluD1 was ablated specifically from striatum [15] and observed a significant reduction in vAChT and vGluT3 expression levels (Fig. 2C, D). We further confirmed the changes in vAChT using immunohistochemistry in WT and GluD1 KO mice tissue. A significant reduction in the number of vAChT-labeled puncta was observed in the dorsal striatum of GluD1 KO mice (Fig. 2E, F). We also examined whether GluD1 may regulate cholinergic terminals in the central amygdala, a brain region enriched in GluD1 [16], and observed a significant reduction in vAChT but an increase in ChAT expression in the GluD1-enriched central amygdala of GluD1 KO mice (Fig. 2G, H). These findings collectively demonstrate that GluD1 is crucial for maintaining cholinergic synaptic integrity. Next, to determine whether these structural deficits translate into functional impairments at cholinergic synapses, we assessed muscarinic receptor activation- induced plasticity in WT and GluD1 KO mice. Cholinergic receptors were activated by bath application of carbachol (5 µM). At this concentration, carbachol is expected to preferentially bind to muscarinic receptors [26–29]. We focused on NMDA receptor mEPSC given the critical role of GluD1 in NMDA receptor regulation [15, 30]. In WT neurons, carbachol induced a significant reduction in NMDA mEPSC frequency as well as amplitude (Fig. 2I–M). In contrast, GluD1 KO neurons exhibited a significantly lower suppression of mEPSCs frequency and amplitude compared to WT. Quantitative analysis confirmed a significant decrease in both the frequency and amplitude in WT neurons, whereas these effects were significantly reduced in GluD1 KO neurons (Fig. 2K, M). These findings demonstrate that loss of GluD1 impairs muscarinic receptor-mediated modulation of excitatory synaptic transmission, indicating a functional disruption of cholinergic synapses onto MSNs.

### Optogenetic stimulation of CINs reveal a GluD1-dependent ion conductance in MSNs

**Fig. 3:**
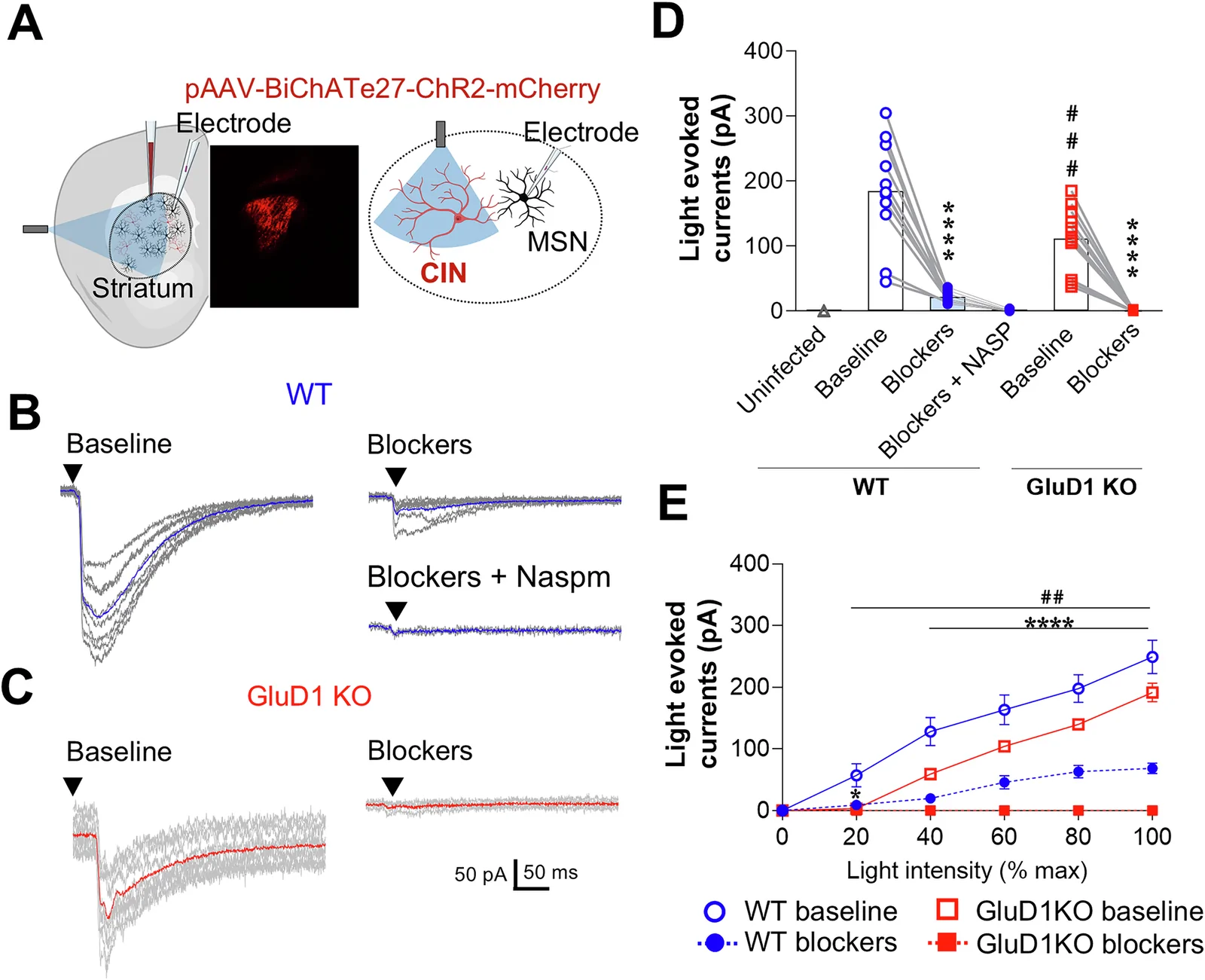
Optogenetic stimulation induces currents sensitive to NASPM and GluD1 deletion. **A** Experimental design. Schematic of viral injection of pAAV-BiChATe27-ChR2-mCherry into the striatum to selectively activate cholinergic interneurons (CINs). Whole-cell recordings were obtained from neighboring MSNs in the dorsal striatum. **B** In WT MSNs light stimulation of CINs evoked robust inward currents under baseline conditions (ACSF). Currents persisted in the presence of glutamatergic (CNQX), GABAergic (picrotoxin), and cholinergic (atropine, mecamylamine) blockers, but were markedly reduced by the subsequent application of NASPM. Representative sweeps are shown in gray with the mean trace in blue. **C** In GluD1 KO MSNs, optogenetic stimulation of CINs evoked robust inward currents under baseline (ACSF) but were abolished by the blocker cocktail (CNQX, picrotoxin, atropine, mecamylamine). Representative traces show light-evoked responses (grey) with the mean trace in red. **D** Quantification. Peak current amplitudes in WT (blue) and GluD1 KO (red) MSNs across baseline, blockers, and blockers + NASPM. In WT MSNs, baseline currents were ~185 ± 9 pA (*N* = 11), which persisted in blockers (~25 ± 9 pA, *N* = 11) but were significantly reduced by NASPM (0 ± 1 pA, *N* = 11). In GluD1 KO MSNs, baseline currents were (~112 ± 6 pA, *N* = 11) and were eliminated by blockers (*N* = 11). ****P = 0.0001. There was also a significant difference between the baseline currents in WT and GluD1 KO, ^###^*P* = 0.0008. Data are presented as mean ± SEM. Statistical comparisons were made using one-way ANOVA with Sidak post-hoc test. **E** Input–output relationship between light stimulation intensity and peak evoked current amplitude in WT and GluD1 KO MSNs. The x-axis represents relative light intensity expressed as a percentage of maximal LED output (arbitrary stimulation units), and the y-axis represents peak light-evoked current amplitude (pA). Increasing optical stimulation resulted in progressively larger inward currents in both groups; however, baseline responses were significantly smaller in GluD1 KO MSNs compared with WT (^##^*P* = 0.0081). The light-evoked responses recorded in WT neurons were significantly reduced by the blocker cocktail (CNQX, picrotoxin, atropine, and mecamylamine; ****P < 0.0001), whereas in GluD1 KO neurons the same blockers abolished the currents (*N* = 6). Data are presented as mean ± SEM. Statistical comparisons were made using one-way ANOVA with Sidak post-hoc test.

We next directly tested the role of GluD1 at cholinergic synapses by optogenetically stimulating striatal cholinergic interneurons (CINs). We conducted patch clamp recordings from MSNs, which are the primary targets of CINs and are enriched in GluD1 [31]. Transduction of CINs with AAV-BiChATe27–ChR2–mCherry [32] enabled light-evoked responses in neighboring MSNs (Fig. 3A). To validate the specificity of viral targeting, we performed immunohistochemistry for ChAT and observed consistent co-localization between ChAT-positive cell bodies and virally infected neurons, confirming selective transduction of CINs (Fig. S2). Both WT and GluD1 KO MSNs showed inward currents following optogenetic activation of CINs (Fig. 3B, C), indicating that cholinergic stimulation recruits postsynaptic conductances regardless of genotype. Interestingly, the amplitude of the response was significantly lower in GluD1 KO (~112 pA) compared to WT MSNs (~185 pA), indicating that ablation of GluD1 reduces the function of cholinergic synapses. Pharmacological manipulation revealed that much of the response was mediated by classical glutamatergic and cholinergic receptors, as the combined application of CNQX, picrotoxin, atropine, and mecamylamine reduced the current by ~85% in WT and abolished it entirely in GluD1 KO MSNs. Strikingly, a small residual current persisted in WT after blockers, which was eliminated by NASPM (100 µM) known to block GluD channels [1, 33–35] (Fig. 3D). Thus, GluD1 appears to mediate an ion conductance at cholinergic synapses. Input–output analysis using increasing light intensities revealed a significant reduction in light-evoked current amplitudes in GluD1 KO MSNs compared to WT controls under both baseline and with blocker cocktail conditions (Fig. 3E). Notably, the residual current observed in WT following receptor blockade increased with stimulus intensity and was absent in GluD1 KO MSNs, further supporting the presence of a GluD1-dependent conductance.

### Acetylcholine induces ion channel currents in MSNs sensitive to NASPM and GluD1 deletion

**Fig. 4:**
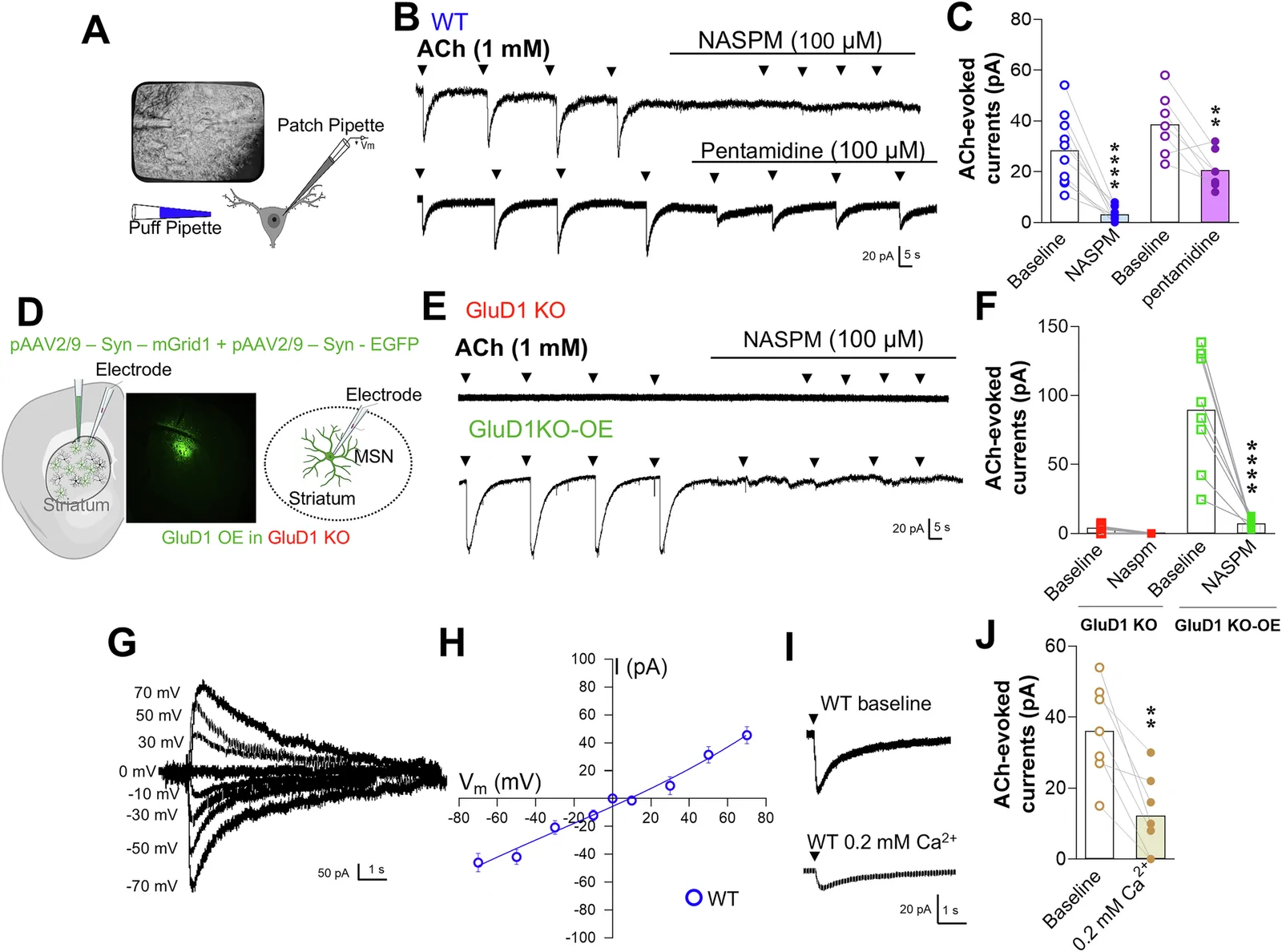
Acetylcholine triggers GluD1-dependent currents in medium spiny neurons. **A** Experimental design. Representative image of a medium spiny neuron (MSN) in the dorsal striatum patched with a recording pipette and held at –70 mV. Acetylcholine (ACh, 1 mM) was applied via a puff pipette placed near the soma. **B** ACh puff application elicited robust inward currents in WT MSNs. **C** The ACh-induced currents in wild-type MSNs had a mean amplitude of ~28 ± 3 pA (*N* = 10) and were significantly reduced by NASPM (100 µM) (to ~3 ± 3 pA, *N* = 10) and a mean amplitude ~38 ± 4.98 pA (*N* = 7) was reduced by pentamidine (100 µM) to (20 ± 5, *N* = 7) **P = 0.0024. **D** Experimental design for rescue. Schematic of GluD1 KO mice injected with pAAV9-Syn-mGrid1 and pAAV9-Syn-EGFP (GluD1KO-OE) into the dorsal striatum; EGFP-labeled MSNs were recorded. **E,** **F** In GluD1 KO MSNs, ACh induced minimal inward currents (~4 ± 3 pA, *N* = 13), ****P = 0.0001. ACh puff application elicited robust inward currents in GluD1KO-OE MSNs (~89 ± 14 pA, *N* = 8) that were significantly reduced by NASPM. **G** Representative traces of GluD1-mediated responses recorded at different holding potentials (–70 mV to +70 mV) upon ACh application via puff pipette. **H** Current–voltage (I–V) relationship of ACh-evoked GluD1 responses. **I,** **J** Ach-induced inward current application was significantly reduced when extracellular Ca^2+^ was lowered to 0.2 mM (*N* = 7) ****P < 0.0001. Data are presented as mean ± SEM. Statistical comparisons were made using one-way ANOVA with Sidak post-hoc test.

Given the localization of GluD1 at cholinergic synapses and the presence of GluD1-dependent ion conductance at cholinergic synapses, we tested whether ACh may also induce GluD1-dependent conductance. Puff application of ACh (1 mM) on MSNs in the presence of mecamylamine, atropine, and CNQX produced inward currents with an average amplitude of ~28 pA (Fig. 4B), which were blocked to ~90% by NASPM and ~50% by pentamidine [1, 4] (Fig. 4C) consistent with a GluD1-mediated conductance. Given that NASPM and pentamidine block ion channel currents through the spontaneously open lurcher mutants of GluD2 and GluD1 [1, 3, 4, 33–36], these blockers have limitations since they also block Ca^2+^-permeable AMPA receptors and NMDA receptors ([37] Podesta et al., Biorxiv). It is, therefore, necessary to further validate the effects of these agents using genetic tools. We further found that GluD1 KO MSNs displayed negligible ACh-induced inward currents under the same conditions and NASPM did not produce significant changes (Fig. 4E, F), confirming that the ACh-evoked responses require GluD1. To further explore this finding, we performed a rescue experiment by reintroducing GluD1 into the MSNs of GluD1 KO mice by stereotaxic injection of AAV-Syn-mGrid1 together with AAV-Syn-EGFP to label infected MSNs (Fig. 4D). Patch-clamp recordings from EGFP-positive MSNs revealed robust ACh-evoked inward currents with an average amplitude of ~90 pA (Fig. 4E). Similar to WT, these currents were abolished by NASPM application, confirming that the restored responses were mediated by GluD1 (Fig. 4F). Notably, the amplitude of the rescued response was substantially larger than that observed in WT, consistent with GluD1 overexpression model. A complementary conditional strategy yielded similar results. Selective deletion of GluD1 using AAV-hSyn-GFP-Cre in GluD1^flox/flox^ mice (Fig. S3A) resulted in a complete loss of ACh-evoked inward currents in infected MSNs, as shown by representative traces (Fig. S3B). In contrast, co-expression of AAV–hSyn–GFP–Cre with AAV–hSyn–mGrid1 (Fig. S3A) restored robust ACh-evoked inward currents, with amplitudes comparable to or exceeding those observed in WT neurons (Fig. S3B–C). Together, these experiments demonstrate that GluD1 is localized at striatal cholinergic synapses and mediates ionic conductance in response to ACh. To further characterize the biophysical properties of the ACh-evoked GluD1-dependent current, we performed voltage-step recordings in MSNs. ACh application elicited inward currents across a range of holding potentials, and the resulting current–voltage (I–V) relationship displayed a near-linear profile with a reversal potential of ~0 mV consistent with a non-selective cation conductance (Fig. 4G–H). Given the previous observations of stabilization of ligand-binding domain dimer interface and facilitation of GluD1 and GluD2 spontaneous currents by extracellular Ca^2+^ [1, 3, 4, 38], we next examined whether extracellular Ca^2+^ affects ACh-evoked current responses. Reducing extracellular Ca²⁺ to 0.2 mM significantly reduced the amplitude of ACh-evoked currents by ~70% consistent with GluD properties (Fig. 4I, J). Together, these findings using pharmacological and genetic tools indicate that the ACh-induced currents show pharmacological and biophysical properties consistent with GluD1 conductances.

### Binding and conformational changes induced by acetylcholine at GluD1

**Fig. 5:**
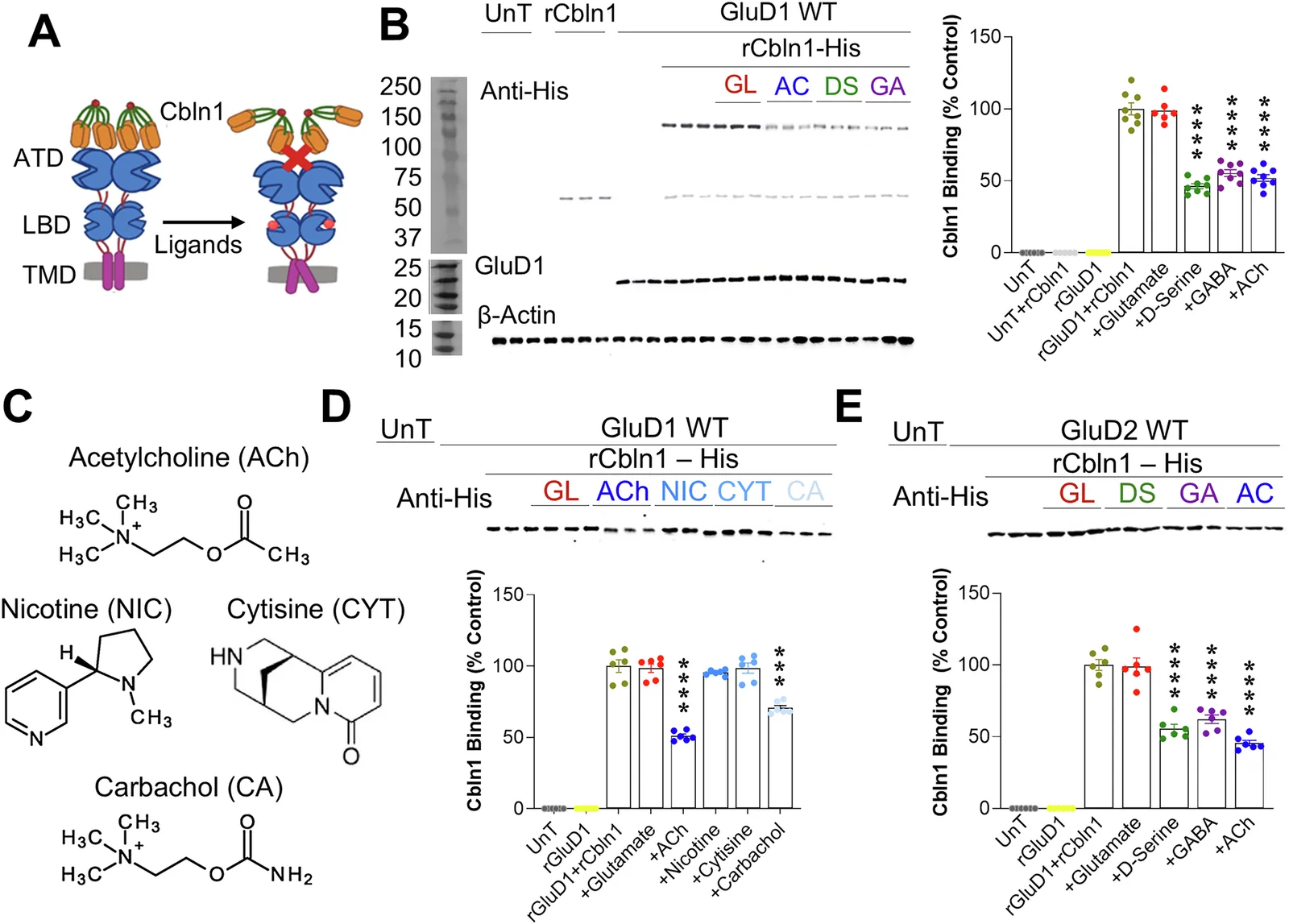
ACh induces conformational changes in GluD1. **A** Schematic model of the principle behind the use of a cell-binding assay as a readout of GluDs ligand binding. Conformational changes induced by ligand-binding to GluDs, overexpressed in HEK293 cells, hinder interaction between Cbln1 and the ATD of GluD1 and reduce Cbln1 binding determined by immunoblotting. **B** Immunoblot analysis of Cbln1 binding in pCAGGS rat GluD1-transfected cell lysates in the presence or absence of glutamate (GL, 1 mM), Acetylcholine (ACh, 1 mM), D-serine (DS, 1 mM), and GABA (GA, 3 mM). Anti-His immunoblotting detected GluD1-dependent Cbln1 binding at ~106 kDa and a non-specific Cbln1 band at ~27 kDa. ACh, D-serine, and GABA reduced GluD1-Cbln1 binding, whereas glutamate had no effect. Total GluD1 levels remained unchanged. Densitometric analysis confirmed a significant reduction in Cbln1 binding in the presence of ACh, D-serine, and GABA (****P < 0.0001). **C** Chemical structures of cholinergic ligands used in this study, including acetylcholine (ACh) and carbachol (CA), cytisine (CYT), and nicotine (NIC). **D** Immunoblot analysis of Cbln1 binding in pCAGGS rat GluD2-transfected cell lysates in the presence or absence of GL, ACh, DS, GA. Anti-His immunoblotting showed GluD2-dependent Cbln1 binding at ~106 kDa and a non-specific band at ~27 kDa. As seen with GluD1, ACh, D-serine, and GABA reduced GluD2-Cbln1 binding, while glutamate did not alter binding. Densitometric quantification showed a significant decrease in binding with ACh, D-serine, and GABA treatment (****P < 0.0001). **E** Cell binding assay performed in the presence of cholinergic agonists, including ACh (1 mM), nicotine (1 mM), cytisine (1 mM), and carbachol (1 mM). Anti-His immunoblotting showed that ACh significantly reduced GluD1-Cbln1 binding. Nicotine and cytisine had no detectable effect, whereas carbachol also reduced binding. Data are presented as mean ± SEM and were analyzed by one-way ANOVA followed by Sidak’s post hoc test (*N* = 6–8).

We next tested whether ACh behaves as a typical ligand for GluD1 and produces current responses in a heterologous expression system. We used a stable cell line similar to that described recently using lentiviral overexpression of human GluD1 in HEK293T cells in which the endogenous GluD1 was deleted [39] (gift from Dr. Cheng Zhu) (Fig. S4A, B). We tested the effect of bath application of ACh in GluD1 overexpressing cells. No currents were observed in these conditions (Fig. S4C). To further evaluate the interaction of ACh with GluD1 we used an assay in which GluD1 ligand-induced conformational changes disrupt Cbln1-GluD1 interaction and was used previously to demonstrate D-serine effect (Fig. 5A) [22]. Using this assay we found that, D-serine and GABA, but not glutamate, reduced the interaction of Cbln1 with GluD1 which is consistent with ligand specificity of GluD1 (Fig. 5B). Importantly, we found that ACh was also able to reduce the interaction between GluD1 and Cbln1, confirming the ability of ACh to bind and induce conformational changes in GluD1. Concentration-response analysis using this assay indicated an IC_50_ of ~3 mM for ACh (Fig. S5). This IC_50_ was similar to that of GABA (Fig. S6). To define the pharmacological profile of this interaction, we evaluated additional cholinergic ligands in the same assay. We used high concentrations (1 mM) of these drugs due to the known low affinity of GluD ligands. Nicotine and cytisine had no effect on Cbln1–GluD1 interaction, whereas carbachol significantly reduced binding suggesting that it induces conformational changes in GluD1 (Fig. 5C). The structural similarity of carbachol with ACh may be responsible for this effect. In contrast, the ring structures in nicotine and cytisine may be incompatible with GluD1 LBD. We next examined whether ACh could also produce conformational changes in GluD2. Using the cell binding assay, we found that D-serine and GABA as well as ACh but not glutamate, induced conformational changes in GluD2 comparable to those observed in GluD1 (Fig. 5D), suggesting that ACh can engage both GluD1 and GluD2.

**Fig. 6:**
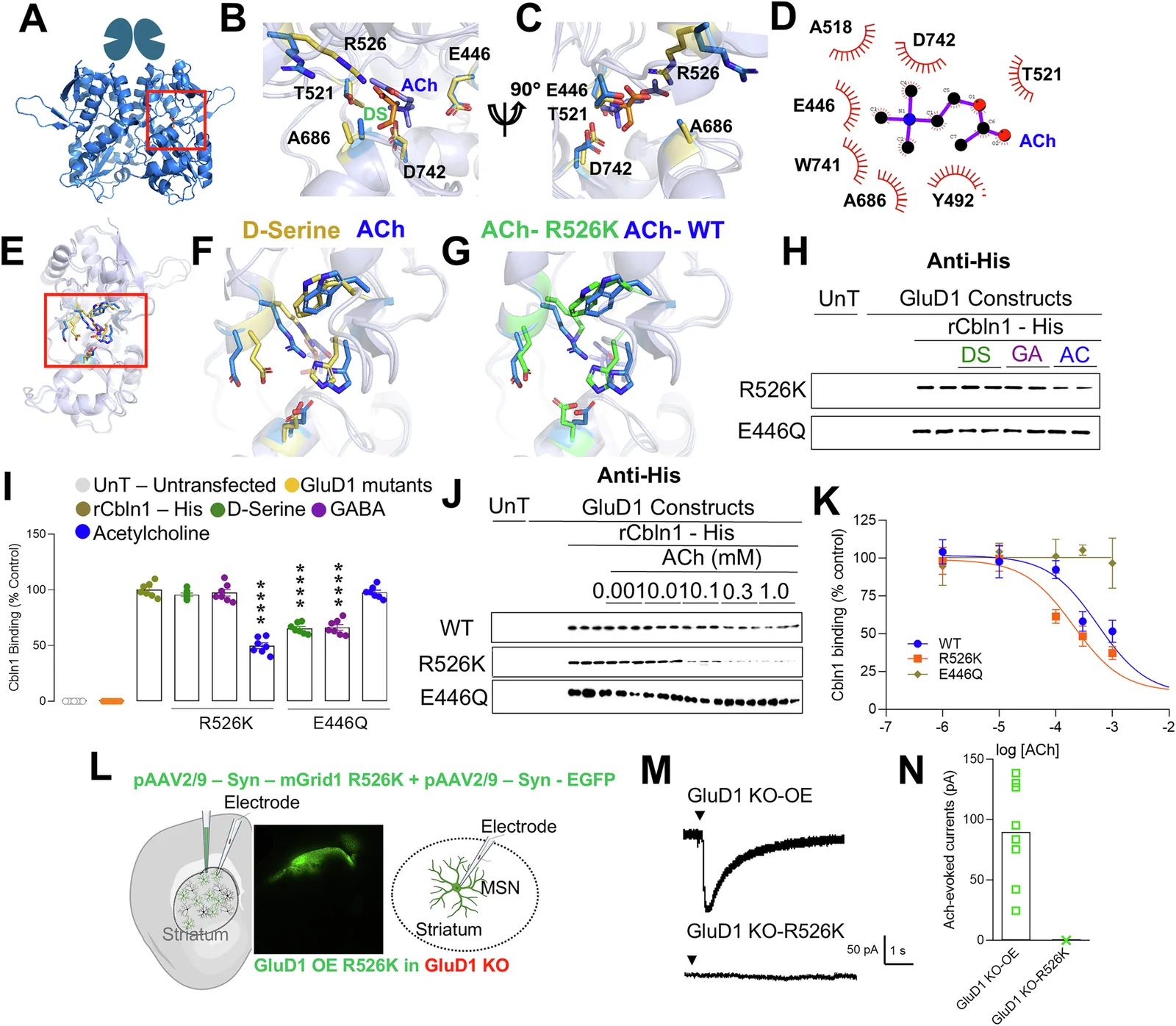
Molecular determinants of ACh binding to GluD1. **A** Illustration of the dimer of LBD of GluD1. X-ray crystal structure of the GluD1 ligand-binding domain (LBD) in complex with D-serine (8BN2 from Piot et al., [3]). **B** MD simulation run derived LBD confirmation with D-serine (yellow) or ACh (blue) in the binding pocket. The key interacting residues interacting with ligands are highlighted. **C** Different view of the MD run with a 90-degree turn. **D** Lig plot diagrams of the ACh interaction with residues in the GluD1 LBD pocket. **E** MD simulation run of ACh (blue) and D-serine (yellow) bound to GluD1 focusing on the interactions of residue R526. **F** Close up of interaction of R526 outside the binding pocket in ACh (blue) or D-serine (yellow) bound state. **G** Interaction of R526 or mutant R526K in ACh bound state in MD simulations. **H**) Immunoblot analysis of Cbln1-His binding onto GluD1 R526K and GluD1 E446Q expressed in HEK293T cells in the presence or absence of D-serine (DS), GABA (GA) or ACh (AC). Anti-His immunoblotting shows Cbln1 band (~106 kDa). **I** Corresponding densitometric analysis of GluD1 R526K confirming the significant reduction in the ACh-treated group, but no changes were observed in the D-serine or GABA treatment (***P < 0.0001). GluD1 E446Q transfected cells showed no changes in GluD1–Cbln1 binding following ACh treatment, but significant reduction was observed upon D-serine and GABA treatment (***P < 0.0001). Data are presented as mean ± SEM, with one-way ANOVA with Sidak’s post-hoc test. **J** Concentration-response analysis of the effect of ACh (0.001–1 mM) on Cbln1 binding in WT GluD1 or mutants. Anti-His immunoblotting revealed concentration-dependent inhibition of GluD1–Cbln1 binding in WT and R526K constructs, while E446Q showed no change in binding across concentrations. **K** Concentration-response curves quantifying ACh-induced disruption of Cbln1 binding across GluD1 WT, R526K and E446Q mutant constructs. **L** Experimental design for GluD1 rescue. Schematic illustrating viral-mediated expression of pAAV9-Syn-mGrid1 R526K together with pAAV9-Syn-EGFP in the dorsal striatum of GluD1 KO mice. Whole-cell recordings were obtained from EGFP-positive MSNs to assess whether GluD1 expression restores ACh-evoked currents. **M** Representative traces showing responses to ACh puff application in MSNs. ACh application elicited robust inward currents in GluD1-overexpressing MSNs, whereas no detectable currents were observed in MSNs expressing the GluD1 R526K mutant. **N** Quantification of ACh-evoked inward current amplitudes. GluD1-overexpressing MSNs exhibited robust ACh-induced currents (89.63 ± 14.91 pA, *N* = 8), whereas MSNs expressing the GluD1 R526K mutant showed no measurable responses (0 pA, *N* = 7).

We next examined the potential structural mechanism by which ACh may bind GluD1. We conducted docking and molecular dynamics simulations (MD) to guide mutational analysis to address this question. ACh was docked to recently described GluD1 LBD structure (PDB 8BN5) [3] and two favorable ACh poses (ACh1 and ACh6), which had similar tertiary amine group location as the primary amines of GABA and D-serine were identified (Fig. S7). In MD, D-serine and GABA were found to maintain configurations as in the crystal structures (Fig. S8, S9 and Table S1-3). During MD for Ach, re-arrangement of the ligand orientation was observed, and the two initially docked poses appeared to converge on a similar binding mode. We, therefore, focused on ACh1 for the remainder of the analysis (Fig. S10). Using the ProLIF trajectory analysis tool for ligand-residue interactions (Table S1-3), some key differences in interactions were noted. Carboxylate groups in D-serine and GABA formed persistent salt bridge with the sidechain of R526 which was completely absent in ACh (Fig. 6B–D; Fig. S8, S9). In contrast, the ester group in ACh strongly interacted with A686. Some common interactions included the amine groups anchored by acidic residues D742 and E446 (for ACh and GABA but not D-serine); A519, Y492, and W741 which provided hydrophobic packing interactions with ligand carbon groups and T521 which stabilizes carboxylate of GABA and D-serine and contacts the ester group of ACh. We further examined the configuration of R526 during the MD and the effect of R526K mutation to better understand its role. R526 was found to interact with H494 and E689 in ACh bound state and appears to stabilize residues along the interface of the two clamshells of LBD (Fig. 6E–G). MD of GluD1R526K with ACh was found to have configuration similar to D-serine at WT GluD1. We next tested the effect of GluD1 mutations based on MD on ACh binding using the cell-binding assay. At GluD1R526K, D-serine and GABA lost the ability to disrupt GluD1-Cbln1 interaction, consistent with the obligatory nature of this residue for ligand binding. In contrast, ACh effect persisted in GluD1R526K (Fig. 6H, I, Fig. S11) and, in fact, ACh showed a leftward shift with an IC_50_ of ~0.2 µM (95% CI: 0.1245–0.3375 µM), (Fig. 6J, K, Fig. S8). Interestingly we found that ACh effect was lost in GluD1E446Q, but the effects of D-serine and GABA persisted, suggesting that this residue is critical for ACh but not D-serine and GABA binding (Fig. 6H, I, Fig. S11).Further concentration-response analysis indicated that the ACh effect on disrupting GluD1-Cbln1 interaction was lost in GluD1E446Q (Fig. 6J, K, Fig. S12). GluD1-Cbln1 interaction in wildtype and mutant GluD1, and the effect of different ligands were also studied in COS-7 cells using immunocytochemistry (Fig. S12). Similar observations were made i.e. residue E446 was critical for ACh induced disruption of Cbln1 binding but not for D-serine and GABA. In contrast R526 residue was critical for D-serine and GABA but not for ACh. These findings collectively reveal a unique binding mode for ACh at the GluD1 LBD, emphasizing E446 as a crucial site for ACh binding and R526 as vital for D-serine and GABA. To determine whether ACh-induced conformational changes translate into functional responses, we examined ACh-evoked currents following reintroduction of WT GluD1 or the GluD1R526K mutant in GluD1 KO MSNs. Re-expression of WT GluD1, but not GluD1R526K, in GluD1 KO neurons restored robust ACh-evoked inward currents (Fig. 6L–N). This finding is surprising given the increase in ACh affinity at GluD1R526K. We hypothesize that the increase in affinity of ACh at GluD1R526K in cell binding assay may signify an increase in desensitization-like phenomenon which may translate into lack of functional ACh-induced currents. This finding however, confirms that ACh interacts with the canonical GluD1 LBD. Finally, using sequence alignment we also explored the conservation of the key Ach-interacting residues in other organisms to understand the evolutionary aspects of this unusual ligand interaction with GluD1 (Fig S13). We primarily focused on organisms that were studied by Rosano et al., who identified GABA-induced current in several bilaterians [40]. Interestingly, a strong conservation of the key residues, particularly those corresponding to E446, T521, R526, and D742 was observed in different organisms. This high degree of conservation supports the idea that the structural core of the LBD required for ligand binding emerged early in evolution and has been maintained. However, some differences were observed in other residues, including T523 and A686. Since GluD homologs from invertebrates are activated by GABA, the occurrence of non-canonical ligand interactions may represent an evolutionary feature of GluDs. In this context, the conservation of GluD1E446 which, based on mutagenesis (E446Q), was found to be critical for ACh but not D-serine and GABA binding, raises the possibility that interaction with ACh, may also be evolutionarily conserved; an issue that can be explored experimentally in the future.

## Discussion

We have previously demonstrated that conditional ablation of striatal GluD1 results in a deficit in cognitive behavioral flexibility [15]. The cholinergic system in the dorsomedial striatum is critical for behavioral flexibility [19–21]. In particular, striatal M1 but not nicotinic receptors [41–43], and NMDA receptors, particularly in the direct pathway MSNs (D1-MSNs) [44, 45], are crucial for reversal learning. Because of the strong behavioral phenotype in mice with striatal GluD1 ablation and the role of GluD1 in the regulation of NMDA receptor function [15, 30], we probed the potential relationship of GluD1 with cholinergic system. Confocal analysis and ultrastructural studies demonstrated that GluD1 is localized to vAChT-labeled and ChAT-labeled cholinergic terminals in the dorsal striatum. In ultrastructural studies GluD1 immunogold particles were found to be associated with immunoperoxidase labeled cholinergic synapses. Importantly, we found that GluD1 was associated with cholinergic synapses in monkey tissue demonstrating the occurrence of this phenomenon across species. In addition to the dorsal striatum, GluD1 was also associated with cholinergic terminals in the IPN region which receives cholinergic input from medial habenula Cbln4-positive neurons [25]. Cholinergic synapses in the dorsal striatum have been found to co-release glutamate or GABA suggesting the presence of two distinct types of cholinergic co-transmitting synapses. [46]. We did not examine whether the cholinergic synapses, at which GluD1 is localized, are glutamatergic or GABAergic. Nonetheless, given the ability of GluD1 to facilitate formation of both excitatory and inhibitory synapses [47] it is possible that GluD1 is localized to either of these synapses.

### Role of GluD1 in the regulation of cholinergic synapses

Ablation of GluD1 produces region specific changes in the formation and maintenance of glutamatergic synapses. For example, in the GluD1 KO prefrontal cortex and CA1 hippocampus excitatory neurotransmission is not reduced [15, 30, 48], whereas in the dorsal striatum and central amygdala mEPSC frequency is reduced to ~50% demonstrating an obligatory nature of GluD1 in the maintenance of excitatory synapses in these regions [15, 16]. GluD1 also facilitates the formation of inhibitory synapses in several brain regions [10, 17]. In addition, GluD1 also regulates the content of AMPA and NMDA receptors at excitatory terminals [15, 30]. Here we demonstrate that loss of GluD1 leads to a significant reduction in the abundance of cholinergic terminals. A robust reduction in the expression of vAChT was observed in the dorsal striatum in both constitutive and conditional GluD1 KO models. Interestingly, in the constitutive GluD1 KO model an increase in ChAT expression was observed which may represent a compensatory change due to developmental deletion of GluD1. GluD1 ablation also led to a reduction in vAChT and an increase in ChAT in the central amygdala a region enriched in GluD1 [16]. In addition to expression changes, functional deficits at cholinergic synapses were also observed. A reduction in carbachol-induced suppression of NMDA receptor mEPSCs and optogenetically stimulated current responses at cholinergic synapses onto MSNs were observed in GluD1 KO, suggesting functional deficit in cholinergic inputs to MSNs. Together these results suggest that loss of GluD1 have both structural and functional effects on cholinergic synapses in multiple brain regions.

### GluD1 mediated ionic conductance in response to ACh in native system

Beyond its role in cholinergic synapse maintenance, optogenetic recordings revealed a residual inward current that persisted despite pharmacological blockade of all known receptor-mediated currents, indicating a possible GluD1-dependent ionic conductance elicited by ACh in the native system. Importantly, the residual inward currents we observed were sensitive to NASPM and were completely absent in GluD1 KO mice, suggesting that GluD1 contributes directly to excitatory ionic conductance at native cholinergic synapses. Consistent with this, ACh puff application elicited inward currents in MSNs even in the presence of cholinergic, AMPA, and GABA_A_ receptor inhibitors, and these currents were blocked by NASPM and pentamidine which are known to block GluD-ionic conductance [1, 3, 4, 33–36]. Notably, this residual current was abolished in GluD1 KO mice and restored upon re-expression of GluD1, strongly implicating GluD1 as the mediator of these ACh-induced ionic responses. However, it should be noted that NASPM and pentamidine can block other glutamate receptors ([37] Podesta et al., Biorxiv), therefore limiting the interpretation of the results using these tools. Although, the use of genetic models partly mitigates these limitations, the development of more specific compounds with increased selectivity for GluDs over other receptor subtypes is needed to further address this issue. We also found that GluD1-dependent currents had a reversal potential of ~0 mV. Previous studies in spontaneously open lurcher mutants suggest double or inward rectification of GluD currents [1, 33–35, 49]. Although we observed some features relevant to this (lower currents at +10 and +30 mV compared to −10 and −30 mV), overall, the IV curve appeared linear. Furthermore, in line with earlier findings [1, 4, 38, 40, 49], we found that GluD1-dependent currents were highly Ca^2+^-dependent. Previous studies have reported tonic GluD1-mediated currents generally dependent on GPCR activation [50, 51], as well as phasic currents triggered by synaptic stimulation or mGluR1/5 activation that are sensitive to NASPM and diminished in GluD1-deficient neurons [52]. Our results extend these findings by suggesting that GluD1 can also contribute to ionic conductance at cholinergic synapses, potentially in response to direct ACh binding. Interestingly, bath application of ACh failed to evoke any measurable currents when GluD1 was expressed in heterologous systems, implying that additional cellular or synaptic components are required to enable this conductance. Recent work indicates that GluD2 can function as an ion channel when incorporated into lipid bilayers but not in cellular environments [6], further supporting the notion that ion channel activity in GluD receptors is highly context dependent.

### ACh binds to GluD1 and induces conformational changes

As an assessment of ACh binding to GluD1 we used an assay we have previously standardized which uses GluD1-Cbln1 interaction as a reporter for ligand binding [22]. We have shown that D-serine binding to GluD1 reduces the interaction between WT GluD1 and Cbln1 and this effect is lost in GluD1R526K mutant. We found that ACh concentration-dependently reduced the binding of Cbln1 to GluD1 in both immunoblotting and immunocytochemistry assay. Furthermore, D-serine and GABA but not glutamate similarly reduced the binding between Cbln1 to GluD1 consistent with ligand specificity of GluD1. Importantly, in this assay although nicotine and cytisine did not produce any effect, carbachol (1 mM) reduced Cbln1-GluD1 binding. These results support the idea that GluD1 has a unique LBD pharmacophore compared to cholinoreceptors and additional analysis is needed to fully understand the structure-activity relationship. Furthermore, it remains to be seen whether low micromolar concentrations of carbachol which activate muscarinic receptors will be capable of modulating GluD1-Cbln1 interaction. We also tested the effect of glutamate, D-serine, GABA and ACh on GluD2-Cbln1 interaction. Interestingly, D-serine, GABA as well as Ach, but not glutamate, reduced GluD2-Cbln1 interaction suggesting conserved LBD features between GluD1 and GluD2. ACh was found to be a low affinity ligand of GluD1 with a EC_50_ of ~3 mM based on cell-binding assay, which is not unusual for GluD ligands like D-serine and GABA which also have low affinity. With regard to the plausibility of ACh being a ligand of GluD1 in physiological condition, it has been found that ACh concentration may reach mM concentrations in the cleft possibly in response to high neuronal activity. Although bulk extracellular ACh levels, measured using microdialysis, generally fall within ~µM range, they do not represent the peak transient concentrations in the cleft following vesicular release [53]. Models of quantal ACh release within the synaptic cleft predict peak concentrations in the sub-millimolar range [54]. Notably, direct time-resolved measurements at individual cholinergic synapses have recorded local ACh transients reaching up to low millimolar levels, with reports of up to ~2.4 mM [55]. In terms of kinetics, ACh transients are expected to be extremely brief (milliseconds) due to the rapid diffusion across the cleft and enzymatic degradation [56]. Together, based on these estimates, it is feasible that GluD1 may be activated in a phasic manner by ACh within the synaptic cleft. In particular GluD1 engagement by ACh may occur during periods of high cholinergic interneuron activity that generate high synaptic ACh transients, consistent with a modulatory mechanism rather than a classical high-affinity receptor interaction. Moreover, the value measured here is an IC_50_ in a Cbln1-GluD1 binding assay, which may not directly correspond to an in vivo functional EC_50_. Future studies will be conducted to fully characterize and understand the activation of GluD1 by ACh release in the synaptic cleft.

The binding orientation of ACh to GluD1 was found to differ from D-serine or GABA. Specifically, ACh did not interact with R526 as observed in D-serine and GABA. Surprisingly, the IC_50_ of ACh was even lower at GluD1R526K suggesting an increase in affinity of ACh with GluD1. This finding is contrary to the known role of the arginine residue in interaction with the carboxylate group of D-serine, glycine and glutamate and the role of this interaction in receptor activation [57]. We further found that E446Q mutation abolished the effect of ACh on GluD1-Cbln1 interaction demonstrating the critical nature of this residue in ACh binding. Despite the leftward shift in binding affinity of ACh at GluD1R526K, the ACh-induced current responses in brain slices were lost in this mutant. We hypothesize that the higher affinity may facilitate the receptor to transition into a desensitization-like state thereby impairing ACh-gated ion channel activity. Additional assessment of the structure-activity relationship in native environment is nonetheless needed. Collectively, these findings uncover a previously unrecognized mechanism by which ACh directly engages GluD1, expanding the repertoire of endogenous ligands capable of modulating GluD1 function and highlighting a potential cholinergic mode of GluD1 regulation in the brain. Furthermore, in relation to ligand-receptor interaction with diverse neurotransmitters, since MD simulations and mutagenesis analysis using cell binding assay suggest that the LBD residues engaged by D-serine and GABA differ from ACh, it is possible that these ligands lead to a different conformational change in the receptor. Considering the trans-synaptic nature of GluD1 complex and the ability of GluD1 C-terminal in regulating AMPA and NMDA receptor expression via intracellular signal transduction [30], it is possible that different conformational changes lead to different outputs. For example, ACh vs D-serine binding may lead to different effects on Cbln1 binding presynaptically or AMPA/NMDA ratio postsynaptically. These effects may then translate to differences in cellular signaling and plasticity.

## Conclusion

GluD1 receptor is bestowed with many unusual characteristics that distinguish it from other iGluRs of which its ability to form trans-synaptic bridge and facilitate both excitatory and inhibitory synapse formation has been of recent interest [8–10]. At a structure-function level, its non-swapped ATD-LBD domain architecture [58] and ability to bind not only D-serine, but also GABA [1, 3], further contribute to its unique properties. Our study demonstrates localization and function of GluD1 at cholinergic synapses. Importantly, we demonstrate that ACh binds and induces conformational changes in GluD1. Given that ACh and cholinoreceptors in the striatum and corticolimbic regions are key regulators of synaptic plasticity, learning, and memory functions [59], and that disruption of these functions occurs in Parkinson’s disease, Alzheimer’s disease and neuropsychiatric disorders [60], our findings raise the possibility that some of these effects may be dependent on GluD1. The discovery of ACh as a ligand for a glutamate receptor opens a new avenue for research in the basic biology and evolution of ion channels as well as drug discovery.

## Supplementary information

Supplementary Data

Supplementary Methods

Resource Table

## Data Availability

Raw data associated with this study are available from the corresponding author upon reasonable request.
