# Supplementary material for: GluD1 is localized at cholinergic synapses and is an acetylcholine receptor
Source: Mol Psychiatry. 2026 Jun 10;31(11):6202–13. doi: 10.1038/s41380-026-03675-4 (PMC13668730; doi:10.1038/s41380-026-03675-4)
Supplement: Supplementary file 1 [file 41380_2026_3675_MOESM1_ESM.docx]

**Supplementary Figures**

**GluD1 is localized at cholinergic synapses and is an acetylcholine receptor**

Poojashree B. Chettiar^1*^, Kishore Kumar S. Narasimhan^1*^, Siddhesh S. Sabnis^1^, Spencer S. Ericksen^2^, Diane Choi^3^, Yoland Smith^3,4^, Shashank M. Dravid^1^

^1^ Department of Psychiatry and Behavioral Sciences, Health Sciences Center, College of Medicine, Texas A&M University, 206, Olsen Blvd, Reynolds Medical Sciences Bldg., College Station, TX 77845, United States, ^2^ Drug Development Core, Small Molecule Screening Facility, University of Wisconsin, Carbone Cancer Center, University of Wisconsin-Madison, ^3^ Emory National Primate Research Center, Emory University, Atlanta, Georgia, ^4^ Department of Neurology, Emory University, Atlanta, Georgia, USA.

* Equal contribution

**Figure S1:** **Specificity of GluD1 antibody and GluD1 localization at cholinergic terminals.**

**(A)** Light micrographs showing lack of striatal GluD1 immunostaining in GluD1 KO and St-GluD1 KO mice confirming the specificity of the GluD1 antibody used in our study. **(B)** Immunofluorescent labeling of GluD1 (green) and vesicular acetylcholine transporter (vAChT, red) in the interpeduncular nucleus reveals frequent colocalization (arrows), suggesting GluD1 expression at cholinergic terminals. **(C-D)** Electron micrographs showing GluD1 immunogold labeling (yellow arrows) at symmetric synapses formed by vAchT+ terminals (immunoperoxidase labeling) in the mouse **(C)** and monkey **(D)** interpeduncular nucleus (N=2 mice; 1 tissue block/mouse; 50 micrographs/block; N=3 monkeys; 1 tissue block/monkey; 50 micrographs/block). Scale bar: 0.5 mm.

**Figure S2:** **pAAV-BiChATe27-ChR2-mCherry selectively expresses in ChAT-positive cholinergic interneurons.** Representative immunofluorescence images showing ChAT (green) and virally expressed pAAV-BiChATe27-ChR2-mCherry (red) in the striatum. The merged image (right) demonstrates strong overlap between ChAT cell body immunoreactivity and BiChAT-mCherry signal (yellow), confirming selective targeting of cholinergic interneurons by the viral construct.

**Figure S3: Validation of ACh-induced current response using conditional deletion and overexpression of GluD1 in the dorsal striatum MSNs.**

**(A)** Experimental design. Stereotaxic injection of AAVs into the dorsal striatum of GluD1^flox/flox^ mice to generate either a conditional knockout (GluD1 cKO) or a rescue condition (GluD1 cKO rescue). For GluD1 deletion, mice were injected with pAAV2/9–hSyn–GFP–Cre. For the rescue condition, mice received pAAV2/9–hSyn–GFP–Cre together with pAAV2/9–Syn–mGrid1, allowing re-expression of GluD1 in the dorsal striatum following Cre-mediated deletion. **(B)** Representative traces showing responses to Ach puff application recorded from MSNs. **(C)** ACh application did not evoke inward currents in GluD1 cKO MSNs (N = 6), whereas robust inward currents were observed in GluD1 cKO rescue neurons (~60 pA, N = 6).

**Figure S4: Lack of current responses by bath application of ACh in GluD1 overexpressing HEK293 cells.**

**(A)** Representative fluorescence images confirming GluD1 expression in a stably GluD1 overexpressing HEK293 cell line (GluD1-OE). **(B)** Immunoblot analysis of whole-cell lysates from the GluD1-OE cell line confirms the presence of Flag-tagged GluD1 protein in the stable line. **(C)** Representative trace from a GluD1-OE cell voltage-clamp recording with bath application of ACh (1 mM). No shift in baseline or detectable inward current were observed (N = 5 cells).

**Figure S5: ACh disrupts GluD1–Cbln1 interactions in a concentration-dependent manner.**

**(A)** Immunoblot analysis of GluD1–Cbln1 binding following ACh treatment. HEK293 cells transfected with GluD1 were incubated with Ach (0.001 – 1 mM), followed by His-tagged Cbln1 (rCbln1-His). Anti-His immunoblotting revealed a dose-dependent reduction in GluD1–Cbln1 interaction. GluD1 (~106 kDa) and Cbln1 (~106 and ~27 kDa) bands are indicated. As a control GluD1 and β-Actin bands are shown. (B) Quantification of the Cbln1 (~106 kDa) band density expressed as percentage of control (no drug) levels. Ach treatment at 0.3 mM and 1 mM resulted in a significant reduction in Cbln1 binding (****P < 0.0001). Data are presented as mean ± SEM from five independent replicates (N = 5).

**Figure S6: GABA disrupts GluD1–Cbln1 interactions in a concentration-dependent manner.**

**(A)** Immunoblot analysis of GluD1–Cbln1 binding following GABA treatment. HEK293T cells transfected with GluD1 were incubated with GABA (0.03 – 30 mM), followed by His-tagged Cbln1 (rCbln1-His). Anti-His immunoblotting revealed a dose-dependent reduction in GluD1–Cbln1 interaction. GluD1 (~106 kDa) and Cbln1 (~106 and ~27 kDa) bands are indicated. **(B)** Quantification of the Cbln1 (~106 kDa) band density expressed as a percentage of control (no drug) levels. GABA treatment at 1.0 mM (**P = 0.0057), 3 mM, 10 mM, and 30 mM (***P < 0.0001) resulted in reduced Cbln1 binding. Data are presented as mean ± SEM from four independent replicates (N = 4).

**Figure S7:** Starting poses for ACh. The ACh poses are rendered as sticks. These represent two docking poses for ACh that serve as starting points for MD analysis in their bound states with the LBD. ACh1 is shown with cyan carbons and ACh6 is shown with dark blue carbons. The tertiary amine groups overlap whereas the ester tails depart in different directions. Both poses have significant overlap with GABA and D-serine (not shown).

**Figure S8: MD simulations of D-serine in GluD1 binding pocket**

**(A)** ProLIF barcodeAnalysis of D-serine for 2000 frames**. (B**) ProLIF barcodeAnalysis of D-serine for 1000 frames **(C)** LigNetwork plots from ProLIF based on interactions of D-serine over final100 ns of production simulation. The frequency of interaction is reflected by the weights of dotted lines.

**Figure S9: MD simulations of GABA in GluD1 binding pocket**

**(A)** ProLIF barcodeAnalysis of GABA for 2000 frames. **(B)** ProLIF barcodeAnalysis of GABA for 1000 frames **(C)** LigNetwork plots from ProLIF based on interactions of GABA over final100 ns of production simulation. The frequency of interaction is reflected by the weights of dotted lines.

**Figure S10: MD simulations of Ach in GluD1 binding pocket**

**(A)** ProLIF barcodeAnalysis of Ach for 2000 frames**. (B)** ProLIF barcodeAnalysis of Ach for 1000 frames **(C)** LigNetwork plots from ProLIF based on interactions of Ach over final100 ns of production simulation. The frequency of interaction is reflected by the weights of dotted lines.

**Figure S11: Effect of GluD1 mutation on ACh-induced disruption of GluD1-Cbln1 interaction.**

**(A)** Immunoblot analysis of GluD1 R526K-Cbln1 binding. HEK293T cells transfected with GluD1 were treated with increasing concentrations of ACh (0.001–1 mM) followed by co-incubation with recombinant His-tagged Cbln1 (rCbln1-His). Anti-His immunoblotting was conducted to assess Cbln1 binding to GluD1. **(B)** Corresponding densitometry quantification of Cbln1 binding. Significant reductions in binding were observed at 0.1 mM, 0.3 mM, and 1 mM (**P < 0.0001). Data are presented as mean ± SEM from five independent replicates (N = 5). **(C)** Immunoblot analysis of GluD1 E446Q–Cbln1 binding following ACh treatment. **(D)** Quantification of ACh-induced disruption of GluD1–Cbln1 binding, showed no changes. Data are presented as mean ± SEM from 5 independent replicates (N=5).

**Figure S12: Ligand-induced inhibition of GluD1–Cbln1 interaction analyzed by immunocytochemistry.**

**(A)** GluD1 WT or mutant overexpressing cos7 cells were incubated with HA-Cbln1 in the presence or absence of ligands (D-serine, 0.3 mM, ACh, 1 mM and GABA 3 mM) and thereafter processed for ICC for immunolabeling of HA. Treatment with D-serine, GABA, or ACh markedly reduced HA intensity in WT and select mutant conditions, indicating disruption of GluD1–Cbln1 binding. **(B–D)** Quantification of HA-positive cells was normalized to control with no ligand (set as 100%) to calculate the percent inhibition of GluD1–Cbln1 interaction. Data are presented as mean ± SEM from three independent experiments with two replicates per group. **(B)** In WT GluD1-expressing cells, all three ligands significantly reduced HA labeling: GABA (63.13 ± 1.69%), ACh (57.16 ± 1.70%), and D-serine (51.58 ± 1.70%), ****P < 0.0001. **(C)** In R526K-expressing cells, ACh significantly reduced HA labeling (59.22 ± 1.31%, ****P < 0.0001), while D-serine and GABA had no effect. **(D)** In E446Q-expressing cells, GABA (51.33 ± 3.82%) and D-serine (53.10 ± 3.82%) significantly reduced GluD1–Cbln1 interaction, while ACh showed no effect. Statistical analysis was performed using Dunnett’s multiple comparisons test.

**Figure S13: Sequence alignment of GluD homologs in different organisms.**

Residues in red were found to be interacting with ACh in MD analysis.

**Table S1:**

|  |  |  | **% interaction in final 100 ns** | | | |
| --- | --- | --- | --- | --- | --- | --- |
| **residue** | **number** | **interaction** | **ACH1** | **ACH6** | **GAB** | **DSR** |
| **GLU** | **446** | Cationic | 72 | 30 | 48 | 0 |
| **GLU** | **446** | HBDonor | 0 | 0 | 48 | 0 |
| **GLU** | **446** | VdWContact | 67 | 48 | 48 | 0 |
| **TYR** | **492** | CationPi | 2 | 0 | 1 | 2 |
| **TYR** | **492** | HBDonor | 0 | 0 | 0 | 4 |
| **TYR** | **492** | Hydrophobic | 96 | 98 | 100 | 0 |
| **TYR** | **492** | VdWContact | 97 | 96 | 60 | 32 |
| HIS | 494 | HBAcceptor | 1 | 0 | 0 | 0 |
| HIS | 494 | VdWContact | 1 | 0 | 2 | 0 |
| **ALA** | **519** | HBDonor | 0 | 0 | 98 | 98 |
| **ALA** | **519** | VdWContact | 79 | 95 | 100 | 100 |
| **ILE** | **520** | Hydrophobic | 12 | 2 | 1 | 0 |
| **ILE** | **520** | VdWContact | 3 | 57 | 26 | 4 |
| **THR** | **521** | HBAcceptor | 0 | 96 | 100 | 100 |
| **THR** | **521** | Hydrophobic | 12 | 1 | 31 | 0 |
| **THR** | **521** | VdWContact | 13 | 98 | 100 | 100 |
| **ARG** | **526** | Anionic | 0 | 0 | 100 | 100 |
| **ARG** | **526** | HBAcceptor | 0 | 0 | 100 | 100 |
| **ARG** | **526** | VdWContact | 0 | 0 | 100 | 100 |
| TYR | 539 | Hydrophobic | 0 | 0 | 1 | 0 |
| TYR | 539 | VdWContact | 0 | 15 | 0 | 0 |
| VAL | 682 | Hydrophobic | 0 | 0 | 0 | 0 |
| SER | 685 | VdWContact | 5 | 0 | 0 | 0 |
| **ALA** | **686** | HBAcceptor | 20 | 0 | 0 | 0 |
| **ALA** | **686** | Hydrophobic | 64 | 96 | 32 | 0 |
| **ALA** | **686** | VdWContact | 34 | 40 | 19 | 3 |
| **VAL** | **687** | Hydrophobic | 0 | 0 | 43 | 0 |
| **VAL** | **687** | VdWContact | 1 | 17 | 1 | 25 |
| PRO | 725 | Hydrophobic | 0 | 0 | 0 | 0 |
| PRO | 725 | VdWContact | 0 | 0 | 0 | 0 |
| **TRP** | **741** | Hydrophobic | 1 | 0 | 47 | 0 |
| **TRP** | **741** | VdWContact | 97 | 100 | 30 | 32 |
| **ASP** | **742** | Cationic | 100 | 100 | 100 | 100 |
| **ASP** | **742** | HBDonor | 0 | 0 | 100 | 100 |
| **ASP** | **742** | VdWContact | 100 | 100 | 100 | 100 |
| VAL | 745 | VdWContact | 6 | 0 | 0 | 0 |
| TYR | 770 | VdWContact | 1 | 0 | 0 | 3 |

**Table S1:** Quantitative interaction table for final 100 ns of production MD simulation. The percent interaction reflects the proportion of frames satisfying the conditions for each interaction. Only contact residues from LBD are shown. The values are colored with deepening blue to reflect more frequent interactions. The key contact residues, as exhibited by the non-trivial occurrence of interactions, are highlighted in bold face and outlined with boxes.

**Table S2:**

|  | **Ach1** | **Ach6** | **GABA** | **D-serine** |
| --- | --- | --- | --- | --- |
| **E446** | 1.0 | 1.0 | 1.0 | 0.0 |
| Y492 | 1.0 | 1.0 | 1.0 | 0.5 |
| A519 | 1.0 | 1.0 | 1.0 | 1.0 |
| I520 | 0.0 | 0.5 | 0.0 | 0.0 |
| **T521** | 0.0 | 1.0 | 1.0 | 1.0 |
| **R526** | 0.0 | 0.0 | 1.0 | 1.0 |
| **A686** | 1.0 | 1.0 | 0.5 | 0.0 |
| V687 | 0.0 | 0.0 | 0.5 | 0.0 |
| W741 | 1.0 | 1.0 | 1.0 | 0.5 |
| ***D742*** | 1.0 | 1.0 | 1.0 | 1.0 |

**Table S2:** Simplified qualitative interaction matrix for final 100 ns of production MD simulation. Interactions were grouped into 3 categories for each residue-ligand pair: 1.0 = strong, 0.5 = weak, 0.0 =none. Strongly interacting residues achieved interactions with a frequency of at least 25% during the final 100 ns production phase. Weakly interacting residues achieved interactions in 10-25% of the frames. Residues producing interactions in less than 10% of frames were considered non-interacting. Boldface residues were identified as having differential interactions across ligands and were considered good candidates for site-directed mutagenesis for functional study. ***D742*** H-bonds only with amines in GABA and D-serine since Ach has tertiary amine (no donors). A cation interaction is made with D742 in all ligand complexes. There may be differences in how the amine binding cavity accommodates the amines of the 3 ligands.

**Table S3:**

| **MD system** | **Starting Lig pose** | **Standard Equilibration parameters** | **Extra Equilibration steps** | **Production MD Duration (ns)** |
| --- | --- | --- | --- | --- |
| 8bn5_gab_ca_rst | 8BN5 xtal | NVT, NPT, 0.1 ns, bb + lig, k=1000 | NPT bb + lig, 5.0 ns, (k=1000)  NPT ca + lig, 5.0 ns, (k=1000)  NPT ca + lig, 5.0 ns (k=200)  NPT ca, 5.0 ns (k=200) | 200  NPT, ca restrained, k=200 |
| 8bn5_dsr_ca_rst | 8BN2 xtal | NVT, NPT, 0.1 ns, bb + lig, k=1000 | NPT bb + lig, 5.0 ns, (k=1000)  NPT ca + lig, 5.0 ns, (k=1000)  NPT ca + lig, 5.0 ns (k=200)  NPT ca, 5.0 ns (k=200) | 200  NPT, ca restrained, k=200 |
| 8bn5_ach_ca_rst | docking pose #1 | NVT, NPT, 0.1 ns, bb + lig, k=1000 | NPT bb + lig, 5.0 ns, (k=1000)  NPT ca + lig, 5.0 ns, (k=1000)  NPT ca + lig, 5.0 ns (k=200)  NPT ca, 5.0 ns (k=200) | 200  NPT, ca restrained, k=200 |
| 8bn5_ach_ca_rst | docking pose #6 | NVT, NPT, 0.1 ns, bb + lig, k=1000 | NPT bb + lig, 5.0 ns, (k=1000)  NPT ca + lig, 5.0 ns, (k=1000)  NPT ca + lig, 5.0 ns (k=200)  NPT ca, 5.0 ns (k=200) | 200  NPT, ca restrained, k=200 |

ns = nanosecond,

NVT = constant number of atoms, volume, and temperature,

NPT = constant number of atoms, pressure, and temperature

ca = alpha carbon restraints on protein,

bb = backbone restraints on protein,

lig = heavy atom (non-hydrogen) restraints on ligand

k=force constant for positional restraint

gab=GABA, dsr=D-serine, ach=acetylcholine

All systems: 300K, PBC, TIP3P water, 150 mM NaCl, 2 temperature control groups: Protein_Ligand and Water_Ions

NVT = constant volume, modified Berendsen thermostat (V-rescale), PME long-range electrostatics, Verlet cutoff-scheme

NPT = constant pressure, modified Berendsen thermostat (V-rescale), Berendsen barostat @ 1 bar, PME electrostatics, Verlet cutoff-scheme

Production MD = NPT, modified Berendsen thermostat (V-rescale), PME, Verlet, Parrinello-Rahman barostat @ 1 bar. If ca restrained, then Berendsen barostat was used instead of Parrinello-Rahman.
