# Supplementary material for: GluD1 is localized at cholinergic synapses and is an acetylcholine receptor
Source: Mol Psychiatry. 2026 Jun 10;31(11):6202–13. doi: 10.1038/s41380-026-03675-4 (PMC13668730; doi:10.1038/s41380-026-03675-4)
Supplement: Supplementary file 2 [file 41380_2026_3675_MOESM2_ESM.docx]

**Supplementary Materials and Methods**

**Animals**

All animal procedures in this study were in line with the National Institutes of Health guidelines for use of animals in research and approved by the Texas A&M University and Emory University Institutional Animal Care and Use Committees (IACUC). Male and female wildtype (WT) and GluD1 knockout (KO) and striatum specific knockout (St-GluD1 KO) mice were used in all experiments [1]. Mice were housed under controlled conditions (22 ± 1 °C; 12 h light/dark cycle) with ad libitum access to food and water. All experimental mice were 2–4 months old and weighed between 20–30 g. The monkey tissue used to collect immuno-EM data came from three rhesus monkeys (2F, 1M; 5-12 years old) from the Emory National Primate Research Center colony. Every effort was made to minimize animal discomfort and stress.

**Antibodies, Chemicals and Drugs**

The antibodies, chemicals, drugs and peptides used in this study are listed in the Resources Table.

**Stereotaxic surgery and AAV injections**

Stereotaxic surgery was conducted as previously described [2] with minor modifications. For virus injection into the dorsal striatum (dSTR), mice were anesthetized with isoflurane (NDC 66794–013-25, Piramal Critical Care, Bethlehem, PA, USA) and placed in a stereotaxic frame (51,733 U, Stoelting, Wood Dale, IL, USA). The skull was exposed, and a small hole was drilled through the skull above the dSTR (anteroposterior (AP): +0.9 mm, mediolateral (ML): ±1.6 mm, dorsoventral (DV): −3 mm. For optogenetic recordings, pAAV-BiChATe27-ChR2-mCherry (Addgene #213830) was injected into the dorsal striatum to enable selective activation of cholinergic interneurons. For GluD1 overexpression experiments, pAAV2/9–hSyn–mGrid1 or pAAV2/9–hSyn–mGrid1 R526K was co-injected with pAAV2/9–Syn–EGFP in WT mice. In GluD1flox/flox mice, pAAV2/9–Syn–GFP–Cre was injected to induce Cre-mediated conditional deletion of GluD1 (GluD1 cKO) and together with pAAV2/9–Syn–mGrid1 for GluD1 overexpression. Viral injections were performed using a NanoFil microliter syringe (World Precision Instruments, Sarasota, FL, USA) fitted with a 33-gauge beveled needle (NF33BV-2, World Precision Instruments). The injection needle was lowered into the dSTR, and virus particles were delivered at a rate of 1 nl/s using a UMP3 microsyringe pump (World Precision Instruments). The needle was left in place at the injection site for an additional 10 min and thereafter slowly withdrawn over a period of 5 min. Incision was sealed with surgical tissue adhesive (3 M Vetbond Tissue Adhesive, MN, USA). Animals were allowed to recover for four to five weeks for viral expression prior to brain slice electrophysiology.

**Immunohistochemistry**

Immunohistochemical analysis was performed as previously described [1]. GluD1 WT and KO mice were transcardially perfused with 4% paraformaldehyde (PFA) in 0.1 M phosphate buffer (PB, pH 7.4) post-fixed overnight in the same fixative at 4°C. Tissues were cryoprotected by sequential immersion in 10%, 20%, and 30% sucrose solutions in 0.1 M PB, then frozen in 2-methyl butane at −45°C to −55°C. Coronal sections 25 μm thick were cut using a Leica CM1900 cryostat and washed in PBS. Sections were blocked in 10% normal goat serum (Jackson Immuno Research, #005-000-121) for 1 h at room temperature, then incubated overnight at 4°C with primary antibodies: guinea pig anti-GluD1 (1:500; Frontier Institute, GluD1C-GP-Af860) and rabbit anti-vChAT (1:500; Novus,) or chicken anti-ChAT (1:500; Aves). After washing, sections were incubated for 2 h at room temperature with AlexaFluor-conjugated secondary antibodies: goat anti-guinea pig AlexaFluor 488 (1:500; Life Technologies, A-11076) and goat anti-rabbit AlexaFluor 594 (1:500; Life Technologies, A-11008) or anti-chicken 594 (1:500; Life Technologies). Labeled sections were mounted using Fluoromount-G (Southern Biotech, Birmingham, AL, USA). Confocal images were acquired using a Nikon Eclipse Ti2-E inverted confocal microscope equipped with an NSPARC scanner. Images of an equivalent region, 2048 × 2048 pixels, were captured using 60x oil immersion. Four to five sections were scanned at several positions at 0.16 μm intervals along the z-axis, and an optical section (8-µm thick) was taken from each tissue section. Puncta numbers were analyzed using NIS-Element image analysis software (Nikon, NY, USA) and were normalized to representative field for presentation. Images from four mice per group were analyzed and plotted.

**GluD1/vAChT double immuno-EM localization**

Tissues from 4 WT mice and 3 monkeys were used in these experiments. All animals were deeply anesthetized with either isoflurane and an overdose of ketamine (100-150 mg/kg, IP) and dormitor (0.1 mg/kg, IP) for mice or pentobarbital (100 mg/kg, IV) for monkeys. Animals were then transcardially perfused with cold oxygenated Ringer’s solution followed by a fixative containing 4% paraformaldehyde and 0.1% glutaraldehyde in phosphate buffer (0.1M, pH 7.4). Afterwards, brains were collected and post-fixed in paraformaldehyde for 24 hours. Tissue blocks were cut in 50 µm-thick coronal sections with a vibrating microtome. To characterize the synaptic relationships between cholinergic terminals and GluD1 in the striatum and interpeduncular nucleus, sections from each region were processed for double immuno-electron microscopy localization of vAChT (immunoperoxidase) and GluD1 (pre-embedding immunogold). After sodium borohydride and cryoprotectant treatments, sections were placed in a pre-incubation solution containing 5% dry milk and PBS for 30 minutes at RT. Sections were then rinsed with TBS containing 0.1% gelatin (TBS-gelatin) and incubated in the primary antibodies solution [1:1000 dilution guinea pig anti vAChT (Synaptic Systems), 1:3000 dilution rabbit anti-GluD1 (Frontiers)], 1% dry milk, and TBS-gelatin for 24 hours at RT. After rinses in TBS-gelatin, sections were incubated in the secondary antibody solution [1.4 nm gold-conjugated goat anti-rabbit IgG (1:100 in TBS-gelatin; Nanoprobes Inc., goat anti-guinea pig biotinylated IgG (1:200 in TBS-gelatin; Vector Laboratories), 1% dry milk, and TBS-gelatin] for 2 hours at RT. Sections were rinsed in TBS-gelatin and 2% sodium acetate buffer before gold particles were silver intensified with the HQ silver kit (Nanoprobes Inc.) for approximately 16-20 minutes. After silver intensification, the avidin biotin complexation (ABC) and 3 ,3”-Diaminobenzidine (DAB) procedures were the same as described in the pre-embedding immunoperoxidase method [3]. After the DAB reaction, sections were rinsed with PB (0.1M, pH 7.4) before they underwent EM processing (osmification, dehydration, and embedding) as described in the pre-embedding immunoperoxidase procedure with the following changes: (1) the sections were fixed in 0.5% osmium tetroxide solution for 10 minutes instead of 20, and (2) sections were stained with 1% uranyl acetate solution for 10 minutes instead of 35.

**Brain slice electrophysiology**

Brain slice electrophysiology was performed as previously described [4, 5] with some modifications. Mice were anesthetized with isoflurane and decapitated, after which brains were rapidly extracted and placed in ice-cold artificial cerebrospinal fluid (aCSF) saturated with 95% O₂ and 5% CO₂. The aCSF was composed of (in mM): 130 NaCl, 24 NaHCO₃, 3.5 KCl, 1.25 NaH₂PO₄, 2.4 CaCl₂, 2.5 MgCl₂, and 10 glucose. Coronal brain slices (250–300 μm) were prepared using a vibratome (Leica VT1200S; Buffalo Grove, IL, USA). Whole-cell patch-clamp recordings were performed from MSNs in the dorsal striatum using an Axopatch 200B amplifier (Molecular Devices, Sunnyvale, CA, USA). Glass pipettes (3–5 MΩ resistance) were filled with an internal solution containing (in mM): 110 mM Cs gluconate, 30 mM CsCl, 5 mM HEPES, 4 mM NaCl, 0.5 mM CaCl_2_, 2 mM MgCl_2_, 5 mM BAPTA, 2 mM Na_2_ATP, and 0.3 mM Na_2_GTP (pH 7.35). QX-314 (2.9 mM) was included to block voltage-gated sodium channels. Cells were voltage-clamped at –70 mV, and only recordings with access resistance below 20 MΩ that changed by less than 20% during the recording were included in the analysis. Signals were filtered at 2 kHz and digitized at 10 kHz using a Digidata 1440A digitizer and pCLAMP 10 software (Molecular Devices). To isolate NMDA receptor–mediated miniature EPSCs (NMDA-mEPSCs), neurons were voltage-clamped at +40 mV in the presence of tetrodotoxin (TTX; 0.5 μM) to block action potentials, picrotoxin (100 μM) to block GABA_A_ receptors, and CNQX (10 μM) to block AMPA/kainate receptors. A stable baseline was established for at least 3 min prior to pharmacological manipulation. Following baseline acquisition, slices were bath-applied with carbachol (5 μM) for 15 min, followed by a 15 min washout period. Drug effects were assessed by comparing mEPSC amplitude and frequency during baseline, drug application, and washout epochs.

For optogenetic recordings, CIN firing was induced by light stimulation (473 nm, 5 ms) through the objective lens. Optogenetic pulses were delivered at 20 s intervals. Agonist-evoked currents were induced by applying 1 mM acetylcholine via a Picospritzer at a pressure of 8 psi for 50 ms for every 60 – 120 s through a borosilicate glass pipette (1–2 MΩ tip resistance). All recordings were performed in the presence of tetrodotoxin (TTX, 0.5 μM), CNQX (10 μM), picrotoxin (100 μM), atropine (4 μM), and mecamylamine (10 μM) to isolate specific receptor-mediated responses. Generally, four stable baseline responses were recorded, followed by bath application of NASPM (100 μM) to assess pharmacological modulation.

**Synaptoneurosome preparation and Immunoblotting**

Synaptoneurosome preparation and western blotting was performed as previously described [6]. Mice were deeply anesthetized with isoflurane and decapitated. Brains were rapidly removed and rinsed in ice-cold artificial cerebrospinal fluid (aCSF) to eliminate residual blood. Coronal brain slices (1 mm thick) were prepared using a brain matrix, and the dorsal striatum region was microdissected. dSTR tissue was homogenized in synaptoneurosome isolation buffer (10 mM HEPES, 1 mM EDTA, 2 mM EGTA, 0.5 mM DTT, 0.5 mM PMSF, 50 μg/mL soybean trypsin inhibitor, 0.25% phosphatase inhibitor cocktails 2 and 3, and 0.25% protease inhibitor cocktail), followed by sonication with three short pulses (FB50 sonicator, Fisher Scientific). Homogenates were filtered twice through three layers of pre-wetted 100-μm nylon mesh (Small Parts Inc.) and once through a 5-μm hydrophilic filter. The final filtrate was centrifuged at 1,000 × g for 10 min at 4°C to collect the synaptoneurosomes pellet. Pellets were resuspended in buffer containing 0.32 M sucrose and 1 mM NaHCO_3_. Protein concentrations were determined as mentioned in immunoblotting above. Equal protein amounts (50 μg per lane) were loaded on 12% SDS-PAGE gels and run at 100 V for 90 min. Proteins were transferred to pre-activated PVDF membranes (GE Healthcare) at 115 V for 1 h 45 min. Membranes were blocked with 5% nonfat milk in TBST (Tris-buffered saline with 0.1% Tween-20) for 1 h at room temperature, followed by overnight incubation at 4°C with GluD1 (1:1000), ChAT (1:1000), vChAT (1:1000), vGluT3 (1:1000), , GAPDH (1:3000) and β-actin (1:3000) primary antibodies. After TBST washing, membranes were incubated with HRP-conjugated secondary antibodies for 1 h at room temperature. Signal detection was performed using SuperSignal™ West Pico PLUS chemiluminescent substrate (Thermo Fisher Scientific, Cat# 34580) and imaged with a ChemiDoc imaging system (Bio-Rad Laboratories). Band intensity was quantified using ImageJ software and normalized to GAPDH/β-actin. Each blot represents an individual animal.

**Cell culture, Transfection**

Cell culture related techniques were done as previously described [5, 6]. COS-7 cells (ATCC Cat# CRL-1651) and HEK293T cells (ATCC Cat# CRL-3216) were cultured using standard procedures and used in low number passages. All cells were maintained in DMEM (Gibco, Cat# C11995500BT) supplemented with 10% fetal bovine serum (FBS, Gibco, Cat# 10099141) and 1% penicillin-streptomycin (100 IU/mL) in a humidified incubator at 37°C with 5% CO₂. For experiments involving GluD1 mutants, HEK293T cells were cultured on 12 mm coverslips in 35 mm dishes and transfected with the following constructs: pCAGGS-rat GluD1 wild-type (WT), pCAGGS-rat GluD1-E446Q and -R526K.

**Cell binding followed by immunoblotting in HEK293T cells**

Quantitative cell binding assays were performed with minor modifications from previously described protocols [5, 7]. Briefly, naïve HEK293T cells were seeded in 6-well plates and transfected at ~40% confluency with either pCAGGS-rat GluD1 WT, pCAGGS-rat GluD2 WT or the mutant constructs E446Q or R526K using polyethylenimine MAX (PEI MAX, Kyfora Bio, Cat# 24765-100) (3 μg DNA construct, DNA and PEI ratio 1:1.5). After 48 hours of transfection and removal of the transfection reagent, cells were treated for 30 minutes with ligands or vehicle control at the indicated concentrations. This was followed by incubation with His-tagged recombinant Cbln1 protein (rCbln1-His, 5 μg/mL) in the continued presence of the respective ligand for an additional 4 hours. Following treatment, the medium was aspirated, and cells were washed three times with PBS. Cell lysates were then collected and processed for immunoblot analysis.

For lysate prep, GentleLys - Native Cell Lysis Buffer (Cube Biotech, Cat# 18906) was added to the cells and incubated on ice for 2 hrs. Following the incubation, the cell lysates were collected by gently patting the plates and aspirating them into a fresh 1.5 mL tube. Protein concentration in the cell lysates was estimated using Pierce™ BCA Protein Assay Kit (Thermo Fisher Scientific, Cat# 23277). Equal amounts of protein from different groups were prepared in a native sample buffer (BioRad Laboratories, Cat# 1610738) and run in a non-reducing polyacrylamide gel. Following separation, the proteins were transferred to polyvinylidene fluoride (PVDF) membranes (BioRad Laboratories, Cat# 1620177) for immunoblotting. The transferred PVDF membranes were blocked using 5% non-fat dry milk powder for 2 hours at room temperature. Following blocking, the membranes were probed using 1:3000; mouse monoclonal anti-his-tag (MBL International Corporation, D-291-3), 1:1000; guinea pig monoclonal anti-GluD1 (Frontiers Institute Co. Ltd., MSFR102510,) and 1:3000 mouse monoclonal anti-βactin (Thermo Fisher Scientific, MA1-140) antibodies. Un-transfected cells treated only with rCbln1-His (without transfection) served as negative controls. The His-Cbln1 band density in the presence of different ligands (glutamate, ACh, GABA, D-serine, nicotine, cytisine and carbachol) was normalized to the band density in no ligand condition.

**Cell binding followed by immunocytochemistry (ICC) in Cos-7 cells**

Cell binding assay in COS-7 cells was performed as described previously [5, 6]. Briefly, COS-7 cells were cultured on a 12 mm coverslip in a 24-well plate. At 40% confluence, the cells were transfected with either pCAGGS-rat GluD1 WT, or the mutant constructs E446Q or R526K (1 μg DNA construct, DNA and PEI ratio 1:1.5). After 48 hours of transfection and removal of the transfection reagent, cells were treated for 30 minutes with ligands or vehicle control. This was followed by incubation with HA-tagged recombinant Cbln1 protein (rHA-Cbln1, 1 μg/mL) in the continued presence of the respective ligand for an additional 4 hours. Following treatment, the medium was aspirated, and cells were washed three times with PBS and processed for ICC. Cells were fixed with 4% paraformaldehyde for 15 mins followed by washing with PBS and incubation with 3% bovine serum albumin (BSA) for 1 hour at room temperature to block nonspecific binding. Then cells were treated with anti-HA primary antibody (Cat# 2367S, Cell Signaling Technologies, Danvers, MA, USA) overnight at 4°C, followed by three PBS washes. Subsequently, the cells were incubated with an anti-mouse secondary antibody conjugated to Alexa Fluor488 (Cat# A21202, Thermo Fisher Scientific, Waltham, MA, USA) for 1 h, followed by three PBS washes. Finally, the coverslips were mounted on glass slides using Fluoromount-G (Cat# 0100-01, SouthernBiotech, Birmingham, AL, USA), and fluorescent images were captured using a Nikon Ti2 confocal microscope. Following the same ICC procedure, the surface expression of GluD1 or Flag was determined in the stable cell lines.

**MD Simulations**

As a baseline, 200 ns molecular dynamics (MD) simulations were performed for GABA and D-serine in their respective holo (ligand-bound, closed clamshell) conformations of the GluD1 ligand-binding domain (LBD). Simulations were conducted under constant temperature and pressure (NPT) conditions in explicit TIP3P water. To maintain the closed conformation of the LBD throughout the production phase, positional restraints were applied to the protein Cα atoms following equilibration. To examine the potential binding of ACh to GluD1-LBD, ACh was docked into the holo-LBD structure, and two plausible binding modes were selected based on docking results, wherein ACh’s cationic amine group occupied the same pocket observed for the amines of GABA and D-serine in their respective crystallographic complexes. These two ACh binding poses were then used as initial coordinates for MD simulations, following the same protocol used for GABA and D-serine, including a 200 ns NPT production run with α-carbon restraints to preserve the closed clamshell state. For comparative analysis of ligand–protein interactions, interaction fingerprints were generated from each trajectory using ProLIF, a Python-based toolkit for MD trajectory analysis [8]. Default settings were used to track time-resolved non-covalent interactions between the ligands and the LBD across the simulation timescale. The details for MD are as follows:

Preparation of Ligand-Binding Domain Complexes: To investigate ligand binding interactions with GluD1 LBD, holo (ligand-bound, closed clamshell) LBD conformations were generated using experimental structures. Bound-state coordinates for γ-aminobutyric acid (GABA) and D-serine were taken from PDB entries 8BN5 and 8BN2, respectively. A monomer from chain A of 8BN5 was used as the structural reference, and the D-serine-bound chain A of 8BN2 was structurally aligned to this reference to extract the initial coordinates for D-serine. Acetylcholine (ACh) coordinates were obtained from PubChem (CID: 187) in 3D MOL format. Ligand protonation states were assigned using OpenEye’s fixpka tool (QUACPAC 2.2.4.0), resulting in zwitterionic forms for GABA and D-serine and a cationic form for ACh. A single low-energy conformer for ACh was generated using OpenEye’s Omega2 (OMEGA 5.1.0.0), and MMFF94 partial charges were assigned via the molcharge module.

Molecular Docking: Docking of ACh into the LBD was performed using Gnina v1.1 (McNutt et al., 2021). The GABA-bound LBD structure (8BN5, chain A) was used with GABA removed, and the co-crystallized GABA binding site was used to define the search box (via autobox_ligand). Docking exhaustiveness was set to 64, and CNN-based rescoring employed the crossdock_default2018 model. Two plausible binding poses were selected based on CNN scores and pose similarity to known bound amine orientations, particularly the occupation of the same pocket by ACh’s cationic amine group as observed in the GABA and D-serine complexes.

System Setup and Molecular Dynamics Simulations: System preparation and MD simulations were performed using GROMACS 2024.4, following protocols adapted from Lemkul’s tutorials (<http://www.mdtutorials.com/gmx>). All LBD-ligand complexes were embedded in a dodecahedral periodic box filled with CHARMM-modified TIP3P water and 0.15 M NaCl, using a 1 nm padding from the solute. Force field parameters for the protein, water, and ions were derived from CHARMM36 (July 2022) [9], and ligand parameters were obtained from CGenFF v4.0 via the SILCSBIO server [10]. Electrostatics were treated using the Particle Mesh Ewald (PME) method (order = 4, Fourier spacing = 0.16 nm), with a 1.2 nm cutoff for Coulombic and van der Waals interactions, and neighbor searching was performed using the Verlet scheme. All simulations used a 2 fs time step.

Equilibration and Production Runs: Each system underwent energy minimization using steepest descent until maximum force was below 10.0 kJ/mol. Equilibration proceeded in six stages: 0.1 ns NVT at 310 K (V-rescale thermostat, τ<sub>T</sub> = 0.1 ps), with strong restraints (1000 kJ mol⁻¹ nm⁻²) on non-hydrogen protein backbone and ligand atoms, 0.1 ns NPT at 310 K and 1 bar (Berendsen thermostat/barostat, τ<sub>P</sub> = 2.0 ps), with the same restraints, 5 ns NPT with strong restraints on protein backbone and ligand, 5 ns NPT with strong restraints on protein α-carbons and ligand, 5 ns NPT with weak restraints (200 kJ mol⁻¹ nm⁻²) on α-carbons and ligand, 5 ns NPT with weak restraints only on α-carbons. Production simulations of 200 ns were then run under NPT conditions (310 K, 1 bar) with weak restraints on protein α-carbons to preserve the closed clamshell conformation. Coordinate snapshots were saved every 10 ps for downstream analysis.

Trajectory Analysis: Trajectory analysis was performed using the ProLIF Python package [8] to generate interaction fingerprints between ligands and LBD residues over the entire production simulation. Default parameters were used. For reproducibility and transparency, all system input files (e.g., .gro, .topol, .mdp) and output files (e.g., .xtc, .log) are available via a publicly accessible Zenodo repository.

Analysis of LBD pocket conservation in iGluR orthologs: The LBD FASTA sequence was extracted directly from the human GluD1 LBD domain structure used in molecular dynamics (based on 8BN5) using the pdb2fasta utility. This sequence was queried on BLASTp webserver (blast.ncbi.nlm.nih.gov) using default settings except the max sequences retrieved parameter was increased from 100 to 1000 sequences. Output 1000 matched FASTA sequences were then supplemented with reference non-mammalian delta sequences: Crassotrea (Magallana gigas, oyster) XP_011428248.2, Danio rerio (zebrafish) AAI62459.1, Saccoglossus kowalevskii (acorn worm) XP_002732006.1, Acanthaster planci (starfish) XP_022085678.1, Diopisthoporus longitubus (marine worm) c29957_g1_i2. Also added were mammalian Rattus norvegicus (rat) sequences Q63226 GluD2, Q62640 GluD1, and an outlier from the AMPA family P19491 GluA2. The sequences were then used as input for a multiple sequence alignment using the Clustal Omega webserver (<https://www.ebi.ac.uk/jdispatcher/msa/clustalo>), using default settings. The alignment was loaded into JalView v2.11.5.1, the human iGluRD1 LDB sequence was set as the reference sequence, and the alignment was reduced only the LDB domain columns of the alignment using the “Hide insertions” function.

**Statistical analysis**

Experimental design incorporated randomization and blinded analysis to ensure equal group sizes and minimize bias. Sample sizes were determined based on prior studies from our laboratory and published literature using similar experimental paradigms, which consistently yielded sufficient statistical power to detect biologically relevant differences. No formal power analysis was performed. All experiments included at least three biologically independent replicates unless otherwise stated. Group size reflects the number of biologically independent replicates, and all statistical analyses were conducted using these independent values. Prior to analysis, datasets were assessed for normality and homogeneity of variance to determine the appropriateness of parametric testing. Statistical analyses were performed using GraphPad Prism version 8.0 (GraphPad Software, San Diego, CA, USA). Data are presented as the mean ± standard error of the mean (SEM). Comparisons between the two groups were analyzed using unpaired two-tailed t-tests. For comparisons involving more than two groups, one-way analysis of variance (ANOVA) followed by Sidak’s post hoc test was employed. A p-value of <0.05 was considered indicative of statistical significance. For sigmoidal concentration-response curves the data was forced to produce close to full inhibition at an arbitrary 30 mM concentration of ligand.

References

1. Liu J, Shelkar GP, Gandhi PJ, Gawande DY, Hoover A, Villalba RM, et al. Striatal glutamate delta-1 receptor regulates behavioral flexibility and thalamostriatal connectivity. Neurobiol Dis 2020; **137:** 104746.

2. Shelkar GP, Gandhi PJ, Liu J, Dravid SM. Cocaine preference and neuroadaptations are maintained by astrocytic NMDA receptors in the nucleus accumbens. Sci Adv 2022; **8:** eabo6574.

3. Choi D, Pare J, Dravid S, Smith Y. Ultrastructural localization of glutamate delta receptor 1 in the rodent and primate lateral habenula. J Comp Neurol 2025; **533:** e70019.

4. Gawande DY, S Narasimhan KK, Shelkar GP, Pavuluri R, Stessman HAF, Dravid SM. GluN2D subunit in parvalbumin interneurons regulates prefrontal cortex feedforward inhibitory circuit and molecular networks relevant to schizophrenia. Biol Psychiatry 2023; **94:** 297–309.

5. Sabnis SS, S Narasimhan KK, Chettiar PB, Shelkar GP, Dravid SM. D-serine disrupts Cbln1 and GluD1 interaction and affects Cbln1-dependent synaptic effects and nocifensive responses in the central amygdala. Cell Mol Life Sci 2025; **82:** 67–z.

6. Sabnis SS, S Narasimhan KK, Chettiar PB, Gakare SG, Shelkar GP, Asati DG, et al. Intravenous recombinant cerebellin 1 treatment restores signalling by spinal glutamate delta 1 receptors and mitigates chronic pain. Br J Pharmacol 2024; **181:** 1421–37.

7. Rong Y, Bansal PK, Wei P, Guo H, Correia K, Parris J, et al. Glycosylation of cblns attenuates their receptor binding. Brain Res 2018; **1694:** 129–39.

8. Bouysset C, Fiorucci S. ProLIF: A library to encode molecular interactions as fingerprints. J Cheminform 2021; **13:** 72–6.

9. Huang J, MacKerell ADJ. CHARMM36 all-atom additive protein force field: Validation based on comparison to NMR data. J Comput Chem 2013; **34:** 2135–45.

10. Vanommeslaeghe K, Hatcher E, Acharya C, Kundu S, Zhong S, Shim J, et al. CHARMM general force field: A force field for drug-like molecules compatible with the CHARMM all-atom additive biological force fields. J Comput Chem 2010; **31:** 671–90.
