# Supplementary material for: GluD1 is localized at cholinergic synapses and is an acetylcholine receptor
Source: Mol Psychiatry. 2026 Jun 10;31(11):6202–13. doi: 10.1038/s41380-026-03675-4 (PMC13668730; doi:10.1038/s41380-026-03675-4)
Supplement: Supplementary file 3 [file 41380_2026_3675_MOESM3_ESM.docx]

**TABLE S2: KEY RESOURCES USED IN THIS STUDY**

| **Resource Type** | **Specific Reagent** | **Source or reference** | **Identifiers** | **Application** |
| --- | --- | --- | --- | --- |
| **Antibodies** | **Primary Antibodies** | | | |
|  | Mouse monoclonal anti-βactin | Thermo Fisher Scientific | Cat# MA1-140, RRID: AB_2536844 | Immunoblotting |
|  | Rabbit monoclonal anti-Cbln1 | Abcam | Cat# ab1813779;  RRID: AB_2480546 | Immunoblotting |
|  | Mouse monoclonal anti-GAPDH | EMD Millipore | Cat# MAB374, RRID: AB_2107445 | Immunoblotting |
|  | Rat monoclonal anti-VGluT3 | Addgene | Cat# 184216 | Immunoblotting |
|  | Rabbit polyclonal anti-vAchT | Novus Biologicals | Cat# NB100-91348, RRID:AB_1218329 | Immunoblotting/  Immunohistochemistry |
|  | Goat polyclonal anti-ChAT | PhosphoSolutions | Cat# 315-CHAT, RRID:AB_2492055 | Immunoblotting |
|  | Chicken polyclonal anti-ChAT | Aves Labs | Cat# CAT, RRID:AB_2313537 | Immunohistochemistry |
|  | Guinea pig polyclonal anti-GluD1 | Frontiers Institute | Cat# GluD1C-GP Af860, RRID: AB_2571759 | Immunoblotting/  Immunohistochemistry/Immunocytochemistry |
|  | Mouse monoclonal anti-Flag-tag | Millipore Sigma | Cat# F3165, RRID:AB_259529 | Immunocytochemistry/  Immunoblotting |
|  | Rabbit monoclonal anti-HA-Tag | Cell Signaling Technology | Cat# 3724, RRID:AB_1549585 | Immunocytochemistry |
|  | Mouse polyclonal anti-His-Tag | MBL International | Cat# D291-3, RRID:AB_10597733 | Immunocytochemistry/  Immunoblotting |
|  | **Secondary Antibodies** | | | |
|  | Goat anti-guinea pig conjugated to AlexaFluor 488 | Thermo Fisher Scientific | Cat# A-11073, RRID:AB_2534117 | Immunohistochemistry |
|  | Goat anti-mouse conjugated to AlexaFluor 594 | Thermo Fisher Scientific | Cat# A-11032, RRID:AB_2534091 | Immunohistochemistry |
|  | Goat anti-mouse conjugated to AlexaFluor 647 | Thermo Fisher Scientific | Cat# A-21235, RRID:AB_2535804 | Immunohistochemistry |
|  | Goat anti-rabbit conjugated to AlexaFluor 488 | Thermo Fisher Scientific | Cat# A-11008, RRID: AB_143165 | Immunohistochemistry |
|  | Goat anti-rabbit conjugated to AlexaFluor 594 | Thermo Fisher Scientific | Cat# A-11012, RRID: AB_141359 | Immunohistochemistry |
|  | Goat anti-rabbit conjugated to AlexaFluor 647 | Thermo Fisher Scientific | Cat# A-21244, RRID: AB_2535812 | Immunohistochemistry |
|  | Goat anti-rat conjugated to AlexaFluor 594 | Thermo Fisher Scientific | Cat# A-11007,  RRID: AB_10561522 | Immunohistochemistry |
|  | Goat Anti-Guinea pig IgG, HRP Conjugated | EMD Millipore | Cat# AP108P, RRID: AB_92413 | Immunoblotting |
|  | Peroxidase-AffiniPure Goat Anti-Mouse IgG (H+L) | Jackson Immunoresearch Laboratories | Cat# 115-035-003,  RRID: AB_10015289 | Immunoblotting |
|  | Peroxidase-AffiniPure Goat Anti-Rabbit IgG (H+L) | Jackson Immunoresearch Laboratories | Cat# 111-035-003,  RRID: AB_2313567 | Immunoblotting |
| **Chemical Compounds, Drugs and Peptides** | Acetylcholine | Santa Cruz Biotech | Cat# sc-202904 | Electrophysiology/ Immunoblotting/  Immunohistochemistry |
|  | GABA | Hello Bio | Cat# HB0882 | Electrophysiology/ Immunoblotting/  Immunohistochemistry |
|  | D-Serine | Hello Bio | Cat# HB0267 | Electrophysiology/ Immunoblotting/  Immunohistochemistry |
|  | Picrotoxin | Hello Bio | Cat# HB0506 | Electrophysiology |
|  | Tetrodotoxin | Hello Bio | Cat# HB1034 | Electrophysiology |
|  | Mecamylamine Hydrochloride | Millipore Sigma | Cat# 5052070001 | Electrophysiology |
|  | Atropine | Millipore Sigma | Cat# PHR3846 | Electrophysiology |
|  | Carbamoylcholine (Carbachol) | Cayman Chemical | Cat# 14486 | Electrophysiology/ Immunoblotting/ |
|  | Nicotine | Millipore Sigma | Cat# 481975 | Electrophysiology/ Immunoblotting/ |
|  | Cytisine | Millipore Sigma | Cat# 712264 | Electrophysiology/ Immunoblotting/ |
|  | Pentamidine | Millipore Sigma | Cat# 1504900 | Electrophysiology |
|  | NASPM trihydrochloride | Hello Bio | Cat# HB0441 | Electrophysiology |
|  | Recombinant Cerebellin-1 HA (rCbln1) | R&D Systems | Cat# 6934-CB-025 | Immunoblotting  Immunohistochemistry |
| **Commercial Assay or Kit** | Pierce™ BCA Protein Assay Kit | Thermo Fisher Scientific | Cat# 23227 | Immunoblotting  Immunohistochemistry |
|  | SuperSignal™ West Pico PLUS Chemiluminescent Substrate | Thermo Fisher Scientific | Cat# 79654 | Immunoblotting |
| **Cell Lines** | COS-7 | ATCC | Cat# CRL-1651, RRID: CVCL_0224 | Immunohistochemistry |
|  | HEK293T (naïve) | ATCC | Cat# CRL-3216, RRID: CVCL_0063 | Immunoblotting |
|  | HEK293T GluD1-Flag Stable cell line | Gift from Dr. Cheng Zhu | N/A | Electrophysiology  Immunoblotting |
| **Constructs and AAVs** | pCAGGS rat GluD1 | Gift from Dr. Yuzaki | N/A | Electrophysiology  Immunoblotting |
|  | pCAGGS rat GluD1 E446Q | Azenta Lifesciences | Custom synthesis (N/A) | Electrophysiology  Immunoblotting |
|  | pCAGGS rat GluD1 R526K | Home-Made | N/A | Electrophysiology  Immunoblotting |
|  | pCAGGS rat GluD2 | Gift from Dr. Yuzaki | N/A | Electrophysiology  Immunoblotting |
|  | AAV2/9.SYN.DIO.mGluD1 |  | Custom Synthesis (N/A) | Electrophysiology |
|  | AAV2/9.SYN.DIO.mGluD1.R526K |  | Custom Synthesis (N/A) | Electrophysiology |
|  | pAAV_BiCHATe27_ChR2-mCherry | Addgene | Cat# 213830 | Electrophysiology  Immunoblotting |
|  | AAV-hSyn-GFP-Cre | UNC | N/A | Electrophysiology |
|  | AAV 8- hsyn-EGFP | UNC | N/A | Electrophysiology |
| **Organism/**  **Strain** | GluD1 Wildtype and Knockout |  | Gao et al., (2007). | Electrophysiology  Immunoblotting |
|  | C57BL/6 | Jackson Laboratories | Strain #:000664, RRID:IMSR_JAX:000664 | Electrophysiology  Immunoblotting |
| **Software or Algorithm** | BioRender | BioRender | <https://biorender.com/> RRID:SCR_018361) |  |
|  | Graph Pad PRISM 8.0 | GraphPad Software Inc. | <https://www.graphpad.com/> (RRID:SCR_002798) |  |
|  | ImageJ | NIH | <https://imagej.nih.gov/ij/index.html> (RRID:SCR_003070) |  |
|  | Minianalysis | Synaptosoft |  |  |
|  | Nis Elements | NIKON | <https://www.microscope.healthcare.nikon.com/products/software/nis-elements> |  |
|  | Adobe Photoshop | Adobe | https://www.adobe.com/products/photoshop/landpa.html?sdid=FR7NYZM2&mv=search&mv2=paidsearch&ef_id=5fc2de8bb897125cf925b6ed116a988e:G:s&s_kwcid=AL!3085!10!79165045583353!!!!79165251442724!!61304975!1721980582 |  |
|  | PaperPal | Researcher.Life by Editage | <https://paperpal.com/> |  |
